# Investigating genotype–phenotype relationship of extreme neuropathic pain disorders in a UK national cohort

**DOI:** 10.1093/braincomms/fcad037

**Published:** 2023-02-20

**Authors:** Andreas C Themistocleous, Georgios Baskozos, Iulia Blesneac, Maddalena Comini, Karyn Megy, Sam Chong, Sri V V Deevi, Lionel Ginsberg, David Gosal, Robert D M Hadden, Rita Horvath, Mohamed Mahdi-Rogers, Adnan Manzur, Rutendo Mapeta, Andrew Marshall, Emma Matthews, Mark I McCarthy, Mary M Reilly, Tara Renton, Andrew S C Rice, Tom A Vale, Natalie van Zuydam, Suellen M Walker, Christopher Geoffrey Woods, David L H Bennett

**Affiliations:** Nuffield Department of Clinical Neurosciences, University of Oxford, Oxford, UK; Nuffield Department of Clinical Neurosciences, University of Oxford, Oxford, UK; Nuffield Department of Clinical Neurosciences, University of Oxford, Oxford, UK; Nuffield Department of Clinical Neurosciences, University of Oxford, Oxford, UK; NIHR BioResource, Cambridge University Hospitals NHS Foundation, Cambridge, UK; Department of Haematology, University of Cambridge, Cambridge, UK; National Hospital for Neurology and Neurosurgery, University College London Hospitals, London, UK; NIHR BioResource, Cambridge University Hospitals NHS Foundation, Cambridge, UK; Department of Haematology, University of Cambridge, Cambridge, UK; Department of Neurology, Royal Free Hospital, London, UK; Department of Clinical and Movement Neurosciences, UCL Queen Square Institute of Neurology, London, UK; Salford Royal NHS Foundation Trust, Salford, UK; King’s College Hospital NHS Foundation Trust, London, UK; Wellcome Centre for Mitochondrial Research, Institute of Genetic Medicine, Newcastle University, Newcastle upon Tyne, UK; Department of Clinical Neurosciences, University of Cambridge, Cambridge, UK; King’s College Hospital NHS Foundation Trust, London, UK; Great Ormond Street Hospital for Children NHS Foundation Trust, London, UK; UCL Great Ormond Street Institute of Child Health, London, UK; NIHR BioResource, Cambridge University Hospitals NHS Foundation, Cambridge, UK; Department of Haematology, University of Cambridge, Cambridge, UK; Faculty of Biology, Medicine and Health, School of Biological Sciences, Division of Neuroscience and Experimental Psychology, University of Manchester, Manchester, UK; Department of Clinical Neurophysiology, Manchester University NHS Foundation Trust, Manchester Academic Health Science Centre, Manchester, UK; Institute of Life Course and Medical Sciences, University of Liverpool, Liverpool, UK; Department of Neuromuscular Disease, UCL Queen Square Institute of Neurology and the National Hospital of Neurology and Neurosurgery, London, UK; NIHR Oxford Biomedical Research Centre, Oxford University Hospitals Trust, Oxford, UK; Wellcome Centre for Human Genetics, University of Oxford, Oxford, UK; Oxford Centre for Diabetes, Endocrinology and Metabolism, University of Oxford, Oxford, UK; Department of Neuromuscular Disease, UCL Queen Square Institute of Neurology and the National Hospital of Neurology and Neurosurgery, London, UK; King’s College Hospital NHS Foundation Trust, London, UK; Pain Research, Department of Surgery and Cancer, Faculty of Medicine, Imperial College London, London, UK; Pain Medicine, Chelsea and Westminster Hospital NHS Foundation Trust, London, UK; Nuffield Department of Clinical Neurosciences, University of Oxford, Oxford, UK; NIHR Oxford Biomedical Research Centre, Oxford University Hospitals Trust, Oxford, UK; Wellcome Centre for Human Genetics, University of Oxford, Oxford, UK; Oxford Centre for Diabetes, Endocrinology and Metabolism, University of Oxford, Oxford, UK; Great Ormond Street Hospital for Children NHS Foundation Trust, London, UK; UCL Great Ormond Street Institute of Child Health, London, UK; Department of Medical Genetics, Cambridge Institute for Medical Research, University of Cambridge, Cambridge, UK; Addenbrookes Hospital, Cambridge University Hospitals NHS Foundation Trust, Cambridge, UK; Nuffield Department of Clinical Neurosciences, University of Oxford, Oxford, UK

**Keywords:** neuropathic pain, whole genome sequencing, peripheral neuropathy, sodium channels

## Abstract

The aims of our study were to use whole genome sequencing in a cross-sectional cohort of patients to identify new variants in genes implicated in neuropathic pain, to determine the prevalence of known pathogenic variants and to understand the relationship between pathogenic variants and clinical presentation. Patients with extreme neuropathic pain phenotypes (both sensory loss and gain) were recruited from secondary care clinics in the UK and underwent whole genome sequencing as part of the National Institute for Health and Care Research Bioresource Rare Diseases project. A multidisciplinary team assessed the pathogenicity of rare variants in genes previously known to cause neuropathic pain disorders and exploratory analysis of research candidate genes was completed. Association testing for genes carrying rare variants was completed using the gene-wise approach of the combined burden and variance-component test SKAT-O. Patch clamp analysis was performed on transfected HEK293T cells for research candidate variants of genes encoding ion channels. The results include the following: (i) Medically actionable variants were found in 12% of study participants (205 recruited), including known pathogenic variants: *SCN9A(ENST00000409672.1):* c.2544T>C, p.Ile848Thr that causes inherited erythromelalgia, and *SPTLC1(ENST00000262554.2):*c.340T>G, p.Cys133Tr variant that causes hereditary sensory neuropathy type-1. (ii) Clinically relevant variants were most common in voltage-gated sodium channels (Na_v_). (iii) *SCN9A(ENST00000409672.1):*c.554G>A, pArg185His variant was more common in non-freezing cold injury participants than controls and causes a gain of function of Na_V_1.7 after cooling (the environmental trigger for non-freezing cold injury). (iv) Rare variant association testing showed a significant difference in distribution for genes NGF, *KIF1A*, *SCN8A*, *TRPM8*, *KIF1A*, *TRPA1* and the regulatory regions of genes *SCN11A*, *FLVCR1*, *KIF1A* and *SCN9A* between European participants with neuropathic pain and controls. (v) The *TRPA1(ENST00000262209.4):c.515C>T,* p.Ala172Val variant identified in participants with episodic somatic pain disorder demonstrated gain-of-channel function to agonist stimulation. Whole genome sequencing identified clinically relevant variants in over 10% of participants with extreme neuropathic pain phenotypes. The majority of these variants were found in ion channels. Combining genetic analysis with functional validation can lead to a better understanding as to how rare variants in ion channels lead to sensory neuron hyper-excitability, and how cold, as an environmental trigger, interacts with the gain-of-function Na_V_1.7 p.Arg185His variant. Our findings highlight the role of ion channel variants in the pathogenesis of extreme neuropathic pain disorders, likely mediated through changes in sensory neuron excitability and interaction with environmental triggers.

## Teaser

Themistocleous *et al*. report that whole genome sequencing revealed clinically relevant variants in 12% of participants with extreme neuropathic pain disorders. The majority of variants were in ion channels, including new phenotype associations (*SCN9A* p.Arg185His and non-freezing cold injury) and novel gain-of-function variants (*TRPA1* p.Ala172Val and episodic pain).

## Graphical Abstract

**Graphical abstract:**
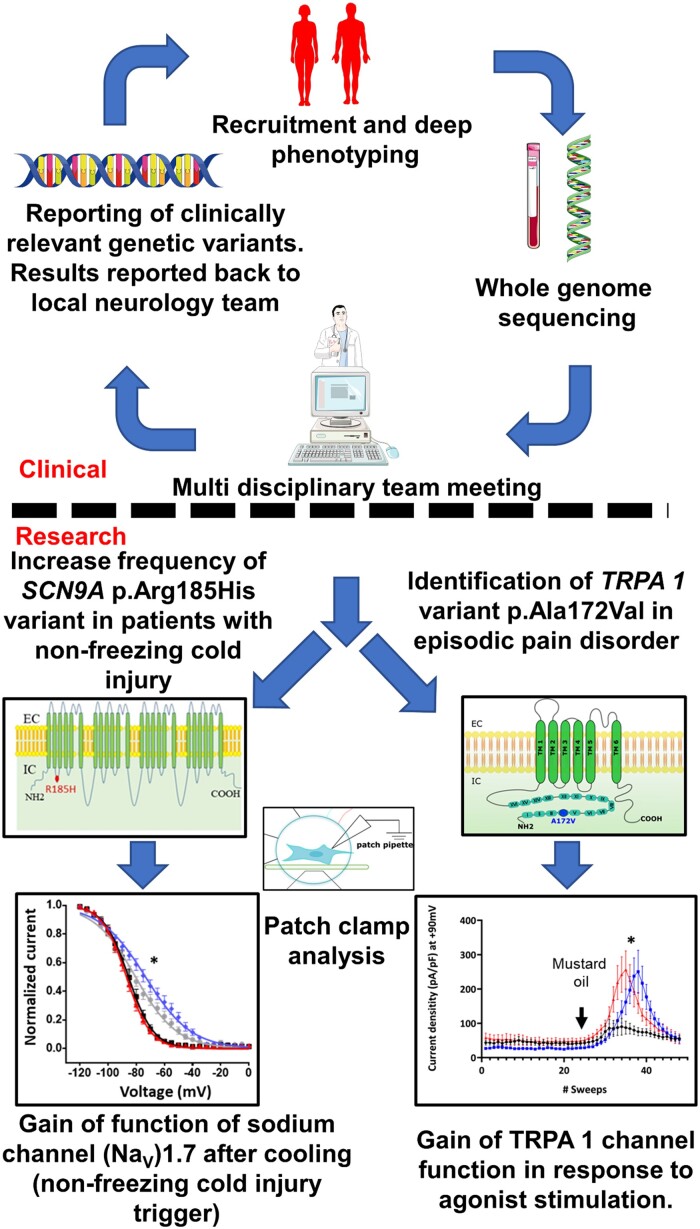

## Introduction

Neuropathic pain occurs as a consequence of a disease or lesion in the somatosensory nervous system.^1^ It affects 6.9–10% of the general population^2^ and has a harmful impact on physical health, psychological health and quality of life.^3^ Understanding the role of genetic factors in neuropathic pain may reveal new pathophysiological mechanisms and is under-explored.^4,5^

Extreme pain phenotypes, caused by rare high-impact genetic mutations, offer insight into fundamental neurobiological mechanisms of pain.^6^ The phenotypes can range from congenital insensitivity to pain^7^ to enhanced pain perception. Different types of mutations in the same gene can cause a spectrum of phenotypes. For example, bi-allelic loss of function mutations in *SCN9A*, the gene encoding the voltage-gated sodium channel (Na_v_) 1.7 and which is highly expressed in peripheral sensory neurons,^8^ causes congenital insensitivity to pain.^9^ In contrast, monoallelic gain-of-function variants in the same gene is associated with pain disorders, which are inherited in a Mendelian fashion. These include inherited erythromelalgia^10^ and paroxysmal extreme pain disorder.^11^

Variants in genes causing Mendelian pain disorders may act as risk factors for common acquired neuropathic pain disorders. For example, *SCN9A* variants are implicated in more common neuropathic pain disorders such as idiopathic small fibre neuropathy^12^ and painful diabetic neuropathy.^13^ The *SCN9A* NM_002977.3:c.3448C>T, p.Arg1150Trp variant modulates risk and severity of pain across different chronic pain disorders.^14^ An environmental trigger that interacts with genes may cause neuropathic pain as some variants are common in the general population. Identification of such gene variants is important in diagnosis, genetic counselling and treatment enabling a stratified approach, such as the use of sodium channel blockers (e.g. lacosamide) in patients with small fibre neuropathy and gain-of-function Na_v_ 1.7 variants.^15^ In some cases, such as inherited erythromelalgia, a personalized management approach can be used.^16,17^ Furthermore, there are rare inherited conditions where direct treatment can arrest progression, such as Fabry’s disease and hereditary transthyretin amyloidosis. In these diseases neuropathic pain is often the first symptom, the disease will progress if untreated and timely genetic diagnosis is essential to initiate appropriate treatment.^18^

The National Institute for Health and Care Research (NIHR) BioResource Rare Disease project applied whole genome sequencing (WGS) to a range of rare diseases, including neuropathic pain disorders.^19^ The aims of our study were to aid the genetic diagnosis of patients with extreme neuropathic pain phenotypes, to determine the prevalence of gene variants associated with neuropathic pain disorders and to understand how the functional changes caused by gene variants relate to clinical presentation and neuropathic pain (Supplementary Fig. 1).

## Methods

### Recruitment and clinical phenotyping of participants

We recruited patients with extreme neuropathic pain phenotypes, both sensory loss and gain, from secondary care clinics in the UK, located in Oxford, London, Salford and Newcastle. Study participants with a history of lifestyle-altering sensory disorder, either pain or loss of sensation, for greater than 3 months were invited to participate. The criteria for clinical case definitions are shown in Table 1.^19^ We excluded patients with a known underlying genetic cause of chronic pain, e.g. Fabry’s disease and *SCN9A* congenital erythromelalgia (genetic pre-screening for these disorders was not mandatory), pregnancy, coincident major psychiatric disorders, poor or no English language skills, patients with documented central nervous system lesions, or patients with insufficient mental capacity to provide informed consent or to complete phenotyping. Description of the clinical phenotyping can be found in the summary NIHR Bioresource paper by Turro *et al*.^19^ and is briefly described here.

**Table 1:** A summary of the neuropathic pain disorders and the key diagnostic criteria used to classify the study participants enrolled

NPD	Diagnostic criteria	Associated phenotypes	Likely inheritance	Known gene (OMIM #)	PMID reference
Congenital insensitivity to pain (including hereditary sensory and autonomic neuropathy type IV, V, VII)	Inability to perceive painful stimuli Other somatosensory modalities may be impaired but the predominant clinical presentation is loss of pain sensibility	Anosmia Autonomic dysfunction Anhidrosis Intellectual impairment	AD, AR	SCN9A (243000), NGF (608654), NTRK1 (256800), SCN11A (615548), PRDM12 (616488), MPV17 (256810), CLTCL1 (601273)	17167479, 17470132, 17597096, 23596073, 19304393, 14976160, 8696348, 24036948, 26005867, 185990, 26068709
Hereditary sensory and autonomic neuropathy type I, II, III	Progressive neuropathies where the presenting or predominant feature is altered sensory function	Autonomic features and motor nerve involvement	AD, AR, X-linked	SPLTLC1 (162400), SPTLC2 (605713), WNK1 (201300), RAB7 (600882), IKBKAP (223900), FAM134B (613115), KIF1A (614213), ATL1 (613708), ATL3 (615632), CCT5 (256840)	11242114, 20920666, 15060842, 12545426, 8102296, 19838196, 21820098, 21194679, 24459106, 16399879
Erythromelalgia	Pain and erythema of the extremities which is exacerbated by warming and relieved by cooling. Initially episodic but may become persistent	Onset by age 20	AD	SCN9A (133020)	14985375
Familial episodic pain syndrome	Severe episodic pain usually localized to the trunk and limbs with no structural cause. Triggers include cold environment, exercise and fasting	Onset usually in childhood Possible family history	AD	TRPA1 (615040), SCN11A (615552)	20547126, 24207120
Small fibre neuropathy	Probable—symptoms in hands and feet consistent with small fibre dysfunction (pain and altered temperature sensibility), clinical signs of small fibre damage (reduced pinprick sensitivity and ability to discriminate warm/cool) and normal nerve conduction studies Definite—symptoms in hands and feet, clinical signs of small fibre damage, normal nerve conduction studies, and altered intra-epidermal nerve fibre density at the ankle and/or abnormal quantitative sensory testing of thermal thresholds at the foot	Autonomic features	AD	SCN9A (133020), SCN10A (615551), SCN11A (615552)	21698661, 23115331, 24207120
Post-traumatic neuropathy	Traumatic nerve injury with clinical evidence of nerve injury in the neuroanatomical distribution of neuropathic pain		N/A	N/A	
Neuropathic pain NOS	Pain with a distinct neuroanatomically plausible distribution; however, no evidence of nerve injury found on clinical examination or specialized investigations		N/A	N/A	

Pattern of inherence for the Mendelian inherited pain disorders, genes (including the OMIM reference) and important PMID references.

NOS, not otherwise specified; NPD, neuropathic pain disorder.

Study participants attended a single appointment that included a clinical assessment, screening for neuropathic pain and specialized investigations to investigate distal symmetrical polyneuropathy. A detailed medical and drug history was taken, followed by a structured upper and lower limb neurological examination to detect clinical signs of distal symmetrical polyneuropathy.^20,21^ DN4 questionnaire was used as a screening tool for neuropathic pain.^22^ Confirmatory tests included nerve conduction studies,^23^ skin biopsy for intra-epidermal nerve fibre density^24,25^ and thermal thresholds in the area of neuropathic pain.^26^ Study participants’ pain was assessed and graded (Supplementary Fig. 2) according to published guidelines.^27^

Whole-blood samples were collected and sent to the NIHR BioResource laboratory in Cambridge. A detailed description of the DNA sequencing, WGS data-processing pipeline and identification of relevant gene variants can be found in Turro *et al*.^19^ and relevant aspects are summarized below.

All participants provided written informed consent in accordance with the Declaration of Helsinki. The study was approved by the East of England Cambridge South national research ethics committee (REC) reference 13/EE/0325.

### Analysis plan

Genetic analysis was completed in two parts. The first analysis was to identify variants of clinical relevance in known pain genes., grouping rare variants in genes for all neuropathic pain phenotypes considering both a targeted panel of pain genes and their promoters and all genes in the human genome that carried rare variants. Two ion channel variants were selected for electrophysiological analysis to investigate their functional impact (Supplementary Fig. 1).

### Clinical reporting of pertinent findings

#### Gene list and transcript selection

A list of genes separated into three tiers were curated at the time of recruitment in 2015 (Tables 2–4) and updated in 2021 (Table 5). The division was based on the strength of evidence for the gene being linked to neuropathic pain.^19^ Only Tier 1 genes (Table 2) were discussed in the multidisciplinary team meetings and considered for clinical reporting.

**Table 2:** A summary of the genes shown to have a causal role in neuropathic pain and included in our analysis

Gene symbol	HGNC database: ID	Chromosomal position	HGNC database: description	Clinical phenotypes	OMIM	PMID reference
**Tier 1 genes**						
FAM134B	25964	5p15.1	Family with sequence similarity 134 member B	HSAN 2B	613115	19838196, 21115472, 24327336
IKBKAP	5959	9q31	Inhibitor of kappa light polypeptide gene enhancer in B-cells, kinase complex-associated protein	Riley Day Syndrome/HSAN 3/Familial Dysautonomia	223900	8102296, 11179021, 11179008
NGF	7808	1p13.1	Nerve growth factor	HSAN 5	608654	14976160, 20978020
NTRK1	8031	1q21-q22	Neurotrophic receptor tyrosine kinase 1	HSAN 4/Congenital insensitivity to pain with anhidrosis	256800	8696348, 11668614, 18077166
PRDM12	13997	9q34.12	PR domain 12	HSAN 8/Congenital insensitivity to pain	616488	26005867, 26975306
RAB7	9788	3q21	RAB7A, member RAS oncogene family	HSAN1/2B	600882	12545426,
SCN9A	10597	2q24.3	Sodium voltage-gated channel alpha subunit 9	Congenital insensitivity to pain, primary erythromelalgia, paroxysmal extreme pain disorder	243000, 133020	17167479, 14985375, 17145499
SCN10A	10582	3p22.2	Sodium voltage-gated channel alpha subunit 10	Painful small fibre neuropathy	615551	23115331, 24006052, 26711856
SCN11A	10583	3p22.2	Sodium voltage-gated channel alpha subunit 11	Familial episodic pain; Insensitivity to pain	615552, 615548	24036948, 24207120, 24776970
SEPT9	7323	17q25.3	Septin 9	Hereditary neuralgic amyotrophy	162100	16186812, 19451530, 21556032
SPTLC1	11277	9q22.31	Serine palmitoyltransferase long chain base subunit 1	HSAN 1	162400	11242114,11242106, 15037712,
SPTLC2	11278	14q24.3	Serine palmitoyltransferase long chain base subunit 2	HSAN 1	605713	12207934, 20920666, 23658386
TTR	12405	18q12.1	Transthyretin	Familial amyloidosis	105210	3011930, 14640030
WNK1	14540	12p13.3	WNK lysine deficient protein kinase 1	HSAN 2	201300	15060842, 18521183, 21625937

At the time of recruitment, 2015, a list of 14 genes with an established causal role in neuropathic pain was curated (Tier 1 genes). The criteria for a gene inclusion were at least three independent families reported with a causal variant in the gene, or two families with additional in vitro functional studies and/or mouse model.

HSAN, Hereditary and Autonomic Sensory Neuropathy; HGNC, HUGO Gene Nomenclature Committee.

**Table 4:** A summary of the genes classified as Tier 3 genes

Gene symbol	HGNC database: ID	Chromosomal position	HGNC database: description	Clinical phenotypes	OMIM	PMID reference
**Tier 3 genes**						
*KCNA1*	HGNC:6218	12p13	Potassium voltage-gated channel subfamily A member 1			20724292
*KCNS1*	HGNC:6300	20q12	Potassium voltage-gated channel modifier subfamily S member 1	No specific Mendelian disorder but the rs734784 allele enhances risk of nerve pain following nerve injury		
*TRPM8*	HGNC:17961	2q37	Transient receptor potential cation channel subfamily M member 8			
*TRPV1*	HGNC:12716	17p13.2	Transient receptor potential cation channel subfamily V member 1			9349813, 9768840
*TRPV2*	HGNC:18082	17p11.2	Transient receptor potential cation channel subfamily V member 2			
*TRPV3*	HGNC:18084	17p13.3	Transient receptor potential cation channel subfamily V member 3			
*TRPV4*	HGNC:18083	12q24.11	Transient receptor potential cation channel subfamily V member 4			
*SCN8A*	HGNC:10596	12q13.1	Sodium voltage-gated channel alpha subunit 8			22493249
*SETDB2*	HGNC:20263	13q14	SET domain bifurcated 2	Mutation identified in this group: HSN/Insensitivity to pain (unpublished data DLB)		

Genes of interest, as determined by expert consensus with little or no published data, were classified as Tier 3 genes.

HSN, hereditary sensory neuropathy; HGNC, HUGO Gene Nomenclature Committee.

**Table 5:** Before exploratory analysis of Tier 2 and 3 genes a contemporaneous search was performed to identify new genes that had been described in the ensuing period from the start of the project to the analysis of data (2015–2021)

Gene symbol	HGNC database: ID	Chromosomal position	HGNC database: description	Clinical phenotypes	OMIM	PMID reference
**Additional genes after contemporaneous search**						
*DNMT1*	HGNC:2976	19p13.2	DNA methyltransferase 1	Neuropathy, hereditary sensory, type IE	126375	21532572, 23365052, 25678562
*Dystonin*	HGNC:1090	6p12.1	Cytoskeleton linker protein	Hereditary sensory and autonomic neuropathy type VI (HSAN6)	614653	22522446
*ZFHX2*	HGNC:20152	14q11.2	Zinc finger homeobox 2	Congenital insensitivity to pain Marsili syndrome (MARSIS)	243000	29253101
*FAAH*	HGNC:50679	1p33	Fatty acid amide hydrolase pseudogene 1	Insensitivity to pain	243000	30929760
*FLVCR1*	HGNC:24682	1q32.3	FLVCR heme transporter 1	Sensory neurodegeneration with loss of pain perception	609144	27923065

These genes were included in Tier 2 and 3 gene analysis.

HSAN, Hereditary and Autonomic Sensory Neuropathy; HGNC, HUGO Gene Nomenclature Committee.

#### Variant filtering to identify variants of clinical relevance

Variants of the 14 Tier 1 neuropathic pain disorders genes were prioritized based on

- Minor Allele Frequency (MAF) in gnomAD < 1/1000 if the variant was not previously described in association with disease, or<25/1000 if the variant was present as disease-causing or questionable disease-causing in Human Gene Mutation Database;
- predicted impact according to Ensembl Variant Effect Predictor were ‘High’, ‘Moderate’ or ‘splice region variant’. Variants with more than three alternate alleles or an internal MAF > 10% were discarded to guard against errors in repetitive regions and prevent potential systematic artefacts.^19^

#### Variant interpretation in multidisciplinary teams meeting

Prioritized variants were assessed by a multidisciplinary team.^19^ Pathogenicity assignment was ascribed according to guidelines of The American College of Medical Genetics.^28^ Variants were classified as pathogenic, likely pathogenic, variant of uncertain significance (VUS), likely benign or benign. Clinically relevant variants were those deemed medically actionable, i.e. variants that could result in specific, defined medical recommendations, and were reported to the referring clinician.

Pathogenic and likely pathogenic variants were deemed clinically relevant.

VUS were deemed clinically relevant if

1. *in vitro* functional studies showed that the variant altered function but the relationship to clinical phenotype was not clear, particularly if a significant gene and environment interaction was suspected; or
2. *in silico* analysis of the variant suggested a significant effect on function as defined by
  - variant position: in a functionally important domain; located in a highly evolutionary conserved section; significant biochemical consequences of amino acid exchange; affecting protein structure;
  - minor allele frequency;
  - concordant pathogenicity through multiple *in silico* algorithms such as SIFT, Polyphen and Align GDVD.

VUS that did not meet the above criteria, likely benign and benign variants were not reported.

### Group-wise rare variant association testing

Genetic association testing in genes carrying rare variants was carried out using the gene-wise approach of the combined burden and variance-component test SKAT-O.^29,30^ The SKAT-O test combines a standard gene burden test that maximizes power under the assumption that all rare variants collapsed in a specific gene region are causal and acting in the same direction towards the phenotype and the sequence kernel association that calculates the weighted sum of squares of the variant score statistics and thus is more robust to the presence of variants with opposing effects. The combined SKAT-O test calculates a linear combination of the burden and variant-component test. Parametric bootstrap was used to resample residuals under the null model. The same resampled bootstrap phenotypes, considering covariates, were used for each gene. The weighting of the linear combination is optimized from the data itself. The Rho statistic indicates the weighting of each test and gives an indication of the causality and directionality of the variants’ effects, with rho = 1 reducing SKAT-O to a burden test (high percentage of causality in the same direction) and a rho = 0 to a SKAT (causal and non-causal variants with opposing directions). Age, sex and the three first Principal Components of genetic variation were used as covariates. Only unrelated individuals were used in the group-wise rare variant analysis. Analysis was done per ethnicity and neuropathic pain clinical phenotype.

#### Data filtering and pre-process

Only participants with neuropathic pain were included for gene-wise rare variant association testing. In total 39 participants were excluded from the original cohort of 205 for the SKAT-O analysis (8 with no neuropathic pain; 2 age not available; 7 sequencing by Genomics England not available as mapped to Grch38 genome assembly and joint genotyping not conducted; 4 as only founders from families included). Exclusions were also based on ethnicity. We only considered 128 Europeans and 38 Africans with neuropathic pain as cases, 4 South Asians and 14 people of ‘Other’ ethnicity were not included. Unrelated individuals from the NIHR Bioresource Rare Diseases cohort, not recruited as a part of neuropathic pain disorders nor the Neurodevelopmental Disorders cohort, were included as controls. Analysis for individuals with European and African ethnic origin was separated. The sample size used was 128 Europeans and 38 Africans as cases versus 5945 and 154 as ethnically matched controls, respectively.

VCF files were normalized and left aligned, and multi-allelic single nucleotide polymorphisms (SNPs) were broken down to bi-allelic using BCF-tools. Only SNPs and indels that were rare in gnomAD (MAF < 0.001), not common in the whole NIHR Bioresources Rare Diseases cohort (MAF < 0.05), and had MAF < 0.05 in cases and controls combined for each phenotype were considered. Variants should have passed the quality filters in the joint genotyping calls and be of high quality with a call rate > 99%, following HWE equilibrium (*P* > 0.05). Alleles should have a high average base call depth (>10) and average genotype quality (>20). Regulatory regions for Tier 1–3 genes were downloaded using the GeneHancer resource form GeneCards. We then selected regions annotated as promoters or promoters/enhancers for the Tier 1–3 genes and collapsed in one group associated with the respective gene. For the panel of Tier 1–3 genes and their promoters, we considered variants with ‘modifier’, ‘moderate’ and ‘high’ impact effects. We further selected variants that were Nonsynonymous or have the EPACTS functional annotations: Essential_Splice_Site, Normal_Splice_Site, Start_Loss, Stop_Loss, Stop_Gain, 5′ UTR and 3′ UTR. For the whole gene set (all genes in the human genome), we only considered variants of ‘moderate’ or ‘high’ Variant Effect Predictor impact on protein-coding genes. Manta and Canvas software packages were used to detect deletions of >50 bp.^19^ Due to the limited sample size and multiple sub-phenotypes, we focused on protein-coding genes and high- and moderate-impact variants for the whole gene set analysis.

Separate analysis was completed for 36 Tier 1–3 genes (72 groups with their respective promoters); functionally validated variants in 3 voltage-gated sodium channels *SCN9A*, *SCN10A* and *SCN11A*^8^; and the whole gene set (∼20 000 genes).

### Functional *in vitro* studies of *SCN9A* p.Arg185His and *TRPA1* p.Ala172Val variants

#### Plasmids and site-directed mutagenesis

Human Na_V_1.7 cDNA was cloned into a modified pcDNA3 expression vector containing downstream IRES and dsRED2 sequences (*SCN9A*-IRES-DsRED). Human β1 and β2 subunits were cloned into pIRES2-AcGFP (SCN1B-IRES-SCN2B-IRES-eGFP).^9^ Human TRPA1 cDNA was cloned into a modified pcDNA3 expression vector containing downstream IRES and dsRED2 sequences (*TRPA1*-IRES-DsRED).^9^ The mutations p.Arg185His, p.Ala172Val and p.Asn855Ser were introduced using QuickChange II XL site-directed mutagenesis kit (Agilent). The clones were sequenced by standard methods.

#### HEK293T cell culture and transfection

Human embryonic kidney HEK-293 T cells were grown in a Dulbecco’s modified Eagle’s culture medium (DMEM/F-12, Invitrogen) containing 10% foetal bovine serum and maintained under standard conditions at 37°C in a humidified atmosphere containing 5% CO_2_. For the study of the *SCN9A(ENST00000409672.1)*:c.554G>A, p.Arg185Hisvariant, cells were transfected using the jetPEI™ transfection reagent (Polyplus-transfection Inc.) with either WT or mutant Na_v_1.7 channel combined with β1 and β2 subunits (2:1 ratio). For the study of *TRPA1(ENST00000262209.4)*:c.515C>T, p.Ala172Val and *TRPA1(NM_007332.3)*:c.2564A>G, p.Asn855Ser variants, cells were co-transfected with pMaxGFP (Amaxa) and either human *TRPA1* wild type (WT) or human *TRPA1*- p.Ala172Val/p.Asn855Ser at a ratio of 1:5 to facilitate visualization of positively transfected cells. The total amount of plasmid DNA transfected was 1.2 μg/μl per 35 mm dish. pMaxGFP positive cells were used as control. Cells were used 36–72 h after transfection. Experiments were performed at room temperature and repeated on three or more separate transfections.

#### Electrophysiology

Voltage clamp experiments were performed on transfected HEK293T cells. Whole-cell patch clamp recordings were conducted using an Axopatch 200B Amplifier, the Digidata 1550B Low Noise Data Acquisition System and the pClamp10.6 software (Molecular Devices). Data were filtered at 5kHz and digitized at 20kHz. Capacity transients were cancelled and series resistance compensated at 70–90% in all experiments. Cells were continuously superfused with an extracellular solution or agonist-containing solutions through a common outlet. Standard extracellular solutions for the patch clamp experiments were used to study the respective *SCN9A*^13^ and *TRPA1*^31,32^ variants.

#### Electrophysiology study of SCN9A p.Arg185His

The extracellular solutions contained (in mM): 140 NaCl,3 KCl, 1 CaCl_2_, 1 MgCl_2_, 10 HEPES, pH 7.3 with NaOH (adjusted to 320 mOsm/l with glucose). Patch pipettes were filled with an internal solution containing (in mM) 140 CsF, 10 NaCl, 1 EGTA, 10 HEPES, pH 7.3 with CsOH (adjusted to 310 mOsm/l with glucose) and had a typical resistance of 2–3 MΩ. Leak currents were subtracted using a P/5 protocol, applied after the test pulse. A holding potential of −100 mV and an intersweep interval of 10 s was used for all the protocols. Measurements were done at 10°C, 20°C and 30°C.

#### Electrophysiology studies of TRPA1 p.Ala172Val and p.Asn855Ser variants

To study the effects of agonist desensitization on channel activation, the extracellular solution contained (in mM): 127 NaCl, 3 KCl, 1 MgCl_2_, 10 HEPES, 2.5 CaCl_2_ and 10 glucose, pH 7.4 with NaOH. Osmolarity was adjusted to 310 mOsm/l with glucose. The intracellular solution contained (in mM): 135 KCl, 2 MgCl_2_, 2 MgATP, 5 EGTA and 10 HEPES, pH 7.4 with CsOH. Osmolarity was adjusted to 300 mOsm/l with glucose.^31^

To study the effects of intracellular calcium in channel modulation, the extracellular solution contained (in mM): 140 NaCl, 4 KCl, 2 CaCl_2_, 1 MgCl_2_, 10 HEPES, pH 7.4 with NaOH. Osmolarity was adjusted to 310 mOsm/l with glucose. The intracellular solution contained (in mM): 130 KCl, 8 NaCl, 2 EGTA, 1 MgCl_2_, 1 CaCl_2_, 4 MgATP, 0.4 Na2GTP, pH 7.4 with CsOH. Osmolarity was adjusted 300 mOsm/l with glucose.

To investigate voltage dependence, currents were recorded during a voltage-step protocol consisting of 400 ms voltage steps to test potentials ranging from −100 to +180 mV, followed by a final invariant step to −75 mV (400 ms) to measure tail currents. The holding potential was set at −0 mV.

The voltage-dependence activation of hTRPA1 p.Ala172Val in response to mustard oil [Allyl isothiocyanate (AITC) a TRPA1 electrophilic agonist] and Menthol (non-electrophilic agonist of TRPA1) was measured. These recordings were performed in a calcium-containing extracellular solution, to preserve agonist desensitization properties.^33^ Perfusion with TRPA1 agonists was performed through a custom-made gravity perfusion system. Immediately after establishing the whole-cell configuration, perfusion was switched to extracellular solution for 2 min before beginning the voltage clamp recording. AITC (Sigma 377430) was dissolved in DMSO (Sigma D2650), and Menthol (Sigma M2772) in ethanol. The maximum final concentration of either DMSO or ethanol did not exceed 0.1%. The effect of TRPA1 agonists on current–voltage curves was measured with a two-voltage-step protocol, as described above. Voltage ramps ranging from −100 to +100 mV for 500 ms, every 5 s, were applied to elucidate the temporal activation of hTRPA1 p.Ala172Val in response to AITC. In this case, the holding potential was set at −70 mV.

Current–voltage curves (*I*–*V* curves) were fitted using a combined Boltzmann and linear ohmic relationship: *I*/*I*_max_ = *G*_max_ (*V*_m_−*E*_rev_)/(1 + exp^(V1/2−Vm)/*k*^). Normalized conductance–voltage curves (activation curves) were fitted with a Boltzmann equation *G*/*G*_max_ = 1/(1 + exp^(V1/2−Vm)/*K*^), where *G* was calculated as follows *G* = *I*/(*V*_m_−*E*_rev_). Steady-state fast inactivation curves were fitted with *I*_T_/*I*_Tmax_ = 1/(1 + exp^−(V1/2−Vm)/*k*^). Tail current-derived voltage-activation curves were fitted to the Boltzmann equation: *I*_T_/*I*_T (Max)_ = 1/(1 + exp^[(Vm−V1⁄2)/*k*]^). In all the equations, *V*_1/2_ represents the half-activation and half-inactivation membrane potentials; *V*_m_ is the membrane potential, *E*_rev_ the reversal potential, *k* the slope factor, *G* the conductance and *I*_T_ the current at a given *V*_m_; *G*_max_ and *I*_Tmax_ are the maximum conductance and current, respectively; *R*_in_ is the fraction of channels that are resistant to slow inactivation. Statistical significance was set at *P* = 0.05 for group comparisons.

### Statistical analysis

Electrophysiology data are presented as mean ± SEM. Statistical analysis for group comparisons included two-way ANOVA (temperature and genotype as a categorical variables) for p.Arg185His and one-way ANOVA (genotype as categorical variable) for p.Ala172Val with Sidak’s multiple comparison test. Statistically significant differences were defined as *P* < 0.05. Frequencies of individual genetic variants were compared across groups using a two-tailed Fisher’s exact test. Significance for gene-wise associations was set to the Bonferroni-adjusted 0.01 threshold for the number of genes considered. Unadjusted *P*-values are reported alongside the significance threshold.

## Results

### Study participants

**Figure 1:**
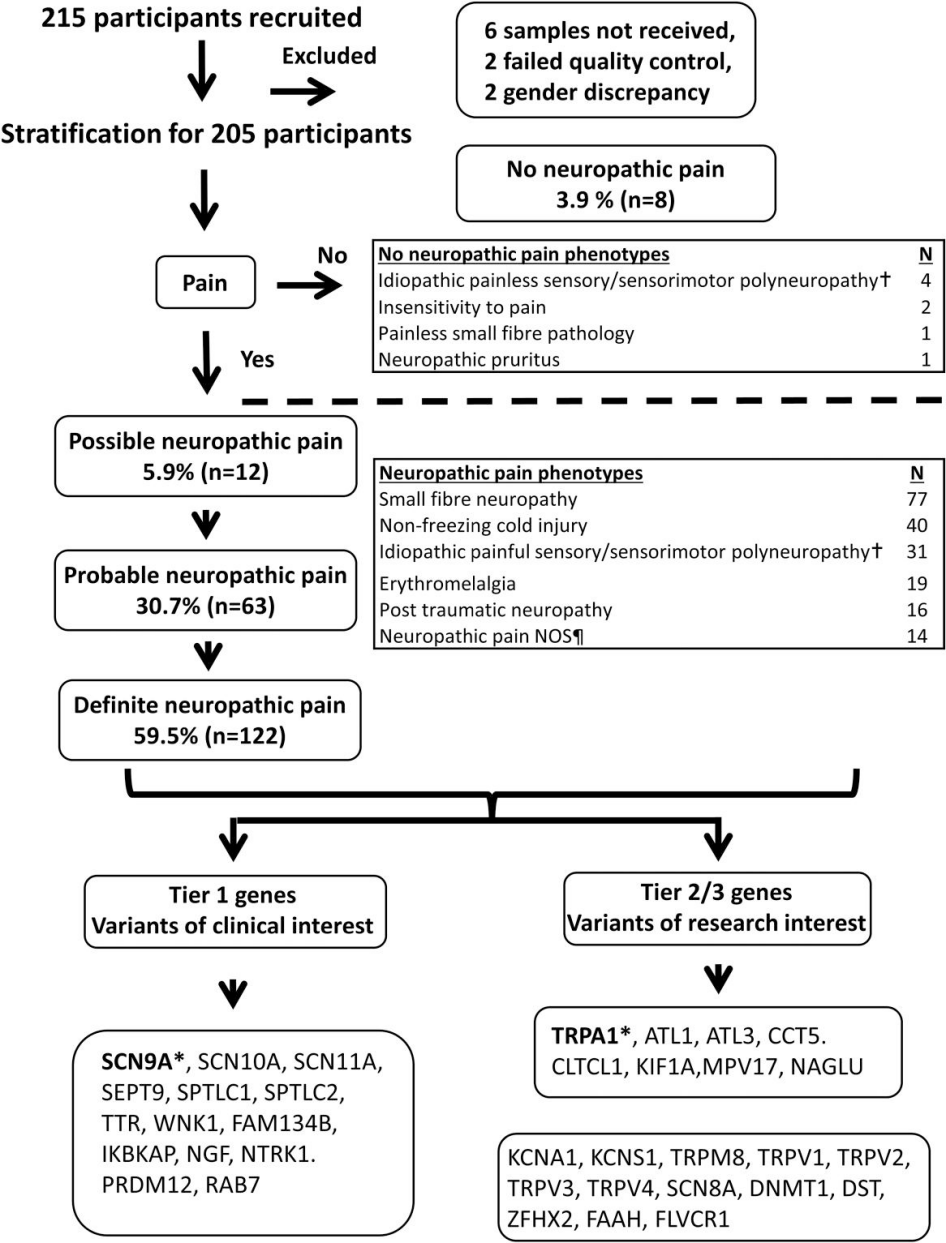
**Flow diagram outlining neuropathic pain grading, study participant recruitment and summary of study participant clinical phenotype.** Ten participants were excluded due to either samples not received, quality control failures or gender discrepancies. ^†^Included cases of longstanding progressive neuropathies where the presenting or predominant feature altered sensory function and an underlying cause could not be identified. ^¶^Include cases of post herpetic neuralgia, episodic pain syndromes, neuropathic pain with plausible neuroanatomical distribution but no abnormalities on examination and specialized tests, leprosy, hereditary neuralgic amyotrophy, type 1 complex regional pain syndrome, Noonan syndrome, injury to left arm (these cases were included due to severe pain which was in excess of the inciting injury). *Genes selected for electrophysiological characterization. NOS, not otherwise specified.

A total of 205 study participants with extreme phenotypes were included (Fig. 1). Age ranged from 4.8 to 84.3 years, and 115 (56.1%) participants were men and 90 (43.9%) were women. In total, 38 participants were recruited of African descent. The majority of participants (90.2%) satisfied the criteria for probable or definite chronic neuropathic pain. Participants with possible neuropathic pain (5.9%) included those with ‘neuropathic-type’ pain (burning, stabbing, electric-like shocks, dysaesthesias) in a neuroanatomically plausible distribution, but with no evidence of nerve injury on clinical examination or specialized investigations. Examples include those with episodic pain syndromes or those with burning pain of the hands and feet with a normal clinical examination and investigations. A group of participants (8, 3.9%) did not experience neuropathic pain, including participants with loss of pain sensation.

### Gene variants reported—Tier 1 gene analysis

After multidisciplinary team discussion, 26 (12.0%) gene variants were categorized as clinically relevant: 3 pathogenic (1.4%), 2 likely pathogenic (0.9%) and 21 VUS deemed relevant for reporting (9.7%). Apart from three participants who declined consent for feedback on genetic testing, all variants were reported to the referring clinician. The results are summarized in Table 6 and Supplementary Table 1.

**Table 6:** List of gene variants that were reported and deemed medically actionable, i.e. variants that result in specific, defined medical recommendations

No	Clinical phenotype	Neuropathic pain grading	Gene	Nucleotide change	Amino acid change	Assigned pathogenicity
1	Erythromelalgia	Definite	SCN9A	c.2543T>C	p.Ile848Thr	Pathogenic
2	Erythromelalgia	Definite	SCN9A	c.2543T>C	p.Ile848Thr	Pathogenic
3	Sensorimotor neuropathy	Probable	SPTLC1	c.399T>G	p.Cys133Trp	Pathogenic
4	Small fibre neuropathy	Definite	SCN10A	c.4984G>A	p.Gly1662Ser	Likely pathogenic
5	Small fibre neuropathy	Definite	SCN11A	c.4628G>A	p.Cys1543Tyr	Likely pathogenic
6	Painful sensory neuropathy	Definite	SPTLC2	c.886A>C	p.Ile296Leu	VUS
7	Non-freezing cold injury	Definite	SCN9A	c.554G>A	p.Arg185His	VUS
8	Non-freezing cold injury	Definite	SCN9A	c.554G>A	p.Arg185His	VUS
9	Non-freezing cold injury	Definite	SCN9A	c.554G>A	p.Arg185His	VUS
10	Non-freezing cold injury	Definite	SCN9A	c.554G>A	p.Arg185His	VUS
11	Non-freezing cold injury	Definite	SCN9A	c.554G>A	p.Arg185His	VUS
12	Non-freezing cold injury	Definite	SCN9A	c.554G>A	p.Arg185His	VUS
13	Small fibre neuropathy	Definite	SCN9A	c.4612T>C	p.Trp1538Arg	VUS
14	Small fibre neuropathy	Definite	SCN9A	c.1445A>G	p.Lys482Arg	VUS
15	Small fibre neuropathy	Probable	SCN9A	c.2215A>G	p.Ile739Val	VUS
16	Small fibre neuropathy	Definite	SCN9A	c.2215A>G	p.Ile739Val	VUS
17	Painful sensory neuropathy	Probable	SCN9A	c.2215A>G	p.Ile739Val	VUS
18	Sensorimotor neuropathy	Definite	SCN9A	c.2215A>G	p.Ile739Val	VUS
19	Small fibre neuropathy	Definite	SCN9A	c.4982A>G	p.Glu1661Gly	VUS
20	Traumatic neuropathy	Probable	SCN9A	c.554G>A	p.Arg185His	VUS
21	Painful Sensory neuropathy	Definite	SCN10A	c.3445G>A	p.Pro1149Met	VUS
22	Small fibre neuropathy	Definite	SCN10A	c.2428G>T	p.Gly810Trp	VUS
23	Painful sensory neuropathy	Definite	SCN10A	c.2737G>A	p.Ala913Thr	VUS
24	Erythromelalgia	Probable	SCN10A	c.968A>G	p.Tyr323Cys	VUS
25	Small fibre neuropathy	Definite	SCN11A	c.2471A>G	p.Glu824Gly	VUS
26	Episodic pain	Possible	SCN11A	c.1730C>T	p.Pro577Leu	VUS

Clinically relevant, medically actionable, variants were reported to the referring clinician. All participants were heterozygous for the relevant variants. All variants were predicted by Ensembl Variant Effect Predictor as missense variants (see Supplementary Table 1 for details of allele frequencies of variants in databases, *in silico* analysis and a list of publications where variants effects on ion channels were studied).

Three participants were diagnosed with pathogenic mutations. Two sisters with severe erythromelalgia, present since childhood, have the same pathogenic variant in *SCN9A* p.Ile848Thr. This variant is not present in control populations, is reported in several inherited erythromelalgia pedigrees and causes gain of function through a hyperpolarizing shift in the voltage dependence of activation.^10,34^ A pathogenic variant in *SPTLC1* p.Cys133Trp was identified in a participant diagnosed with a painful sensorimotor neuropathy and is the commonest pathogenic *SPTLC1* mutation causing hereditary sensory neuropathy type-1 identified in UK patients.^35^

Two participants diagnosed with small fibre neuropathy carried likely pathogenic variants, *SCN10A* p.Gly1662Ser and *SCN11A* p.Cys1543Tyr. This conclusion was based on criteria available at the time of interpretation, low frequency in genetic databases, location in the important region of the channel, high probability of affecting channel function and previous description in small fibre neuropathy^12^; however, we note that this variant is classified as likely benign in ClinVar. The remaining 21 variants reported were classified as VUS, due to our conservative approach in assigning pathogenicity.

**Figure 2:**
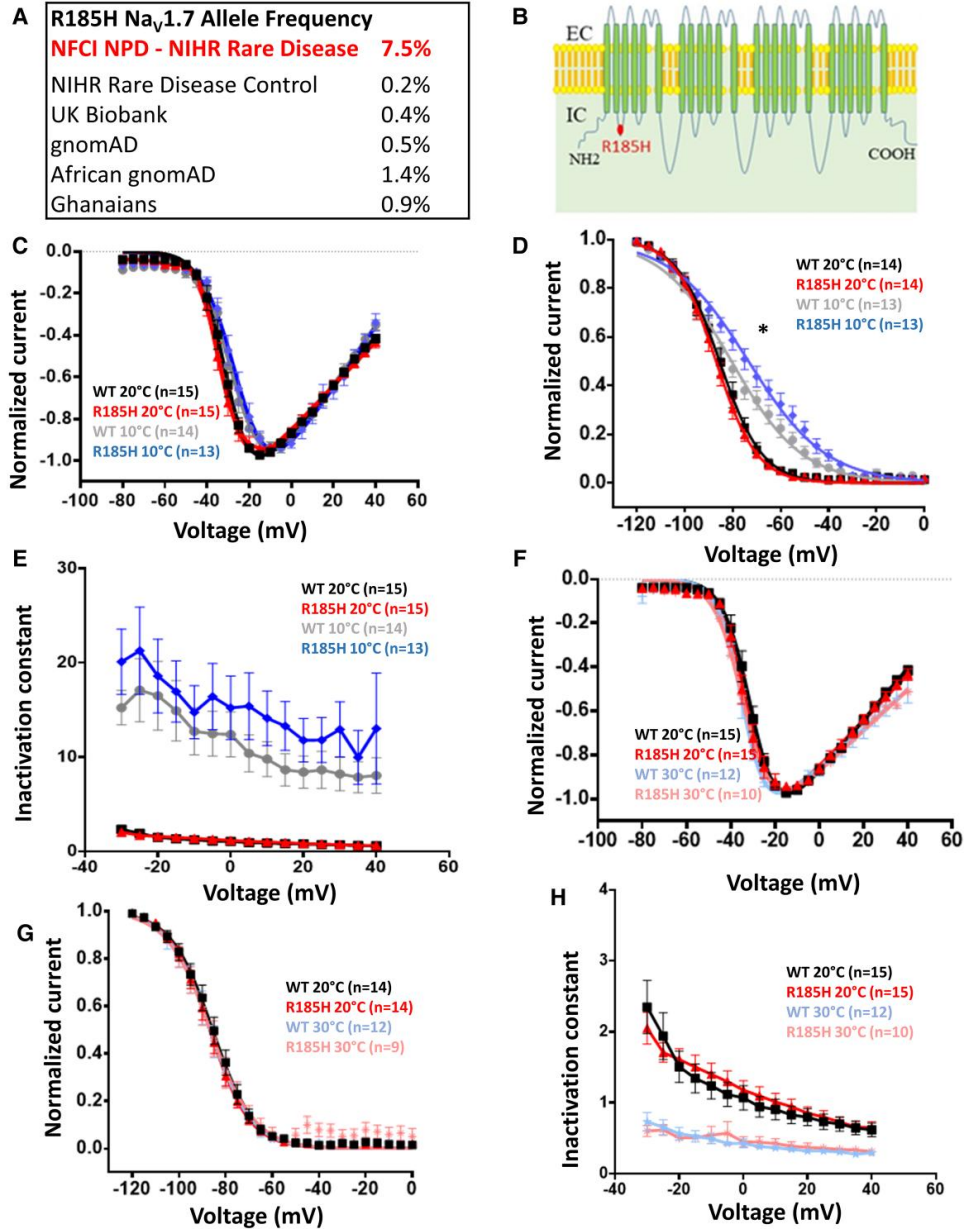
**Description and biophysical characterization of Na_V_1.7 channel p.Arg185His variant, which shows gain of function only at lower temperatures.** (**A**) p.Arg185His is more common in non-freezing cold injury than control populations. (**B**) Schematic of Na_V_1.7 channel topology. R185H is represented with a red dot. (**C**) Normalized peak current–voltage relationship curves for the WT at 20°C (black squares, *V*_1/2_ = −30.7 ± 1.3, *k* = 4.9 ± 0.4, *n* = 15), WT at 10°C (grey dots, *V*_1/2_ = −26.5 ± 2, *k* = 6.6 ± 0.6, *n* = 14), R185H at 20°C (red triangles, *V*_1/2_ = −31.7 ± 1.7, *k* = 5 ± 0.5, *n* = 15), R185H at 10°C (blue diamonds, *V*_1/2_ = −23.8 ± 1.8, *k* = 6.7 ± 0.5, *n* = 13). R185H was not significantly different when compared with WT (*P* > 0.05, two-way ANOVA with Sidak’s multiple comparison test, temperature and genotype are categorical variables). Currents elicited from a holding potential of −100 mV to different test pulse potentials (50 ms) ranging from −80 to 40 mV in 5 mV increments. A holding potential of −100 mV and an intersweep interval of 10 s was used for all the protocols. (**D**) Steady-state fast inactivation curves for the WT at 20°C (black squares, *V*_1/2_= −85.8 ± 1.8, *k* = 7.5 ± 0.6, *n* = 14), WT at 10°C (grey dots, *V*_1/2_ = −80 ± 1.8, *k* = 13.7 ± 0.6, *n* = 13), R185H at 20°C (red triangles, *V*_1/2_= −86.9 ± 1.5, *k* = 7.6 ± 0.3, *n* = 14), R185H at 10°C (blue diamonds, *V*_1/2_= −73.1 ± 2.4, *k* = 14.7 ± 0.6, *n* = 13). R185H significantly different when compared with WT at 10°C (*P* < 0.05, two-way ANOVA with Sidak’s multiple comparison test, temperature and genotype are categorical variables). Currents elicited with test pulses to −10 mV following 500 ms inactivating prepulses. (**E**) Open-state fast inactivation kinetics for the WT at 20°C (black squares, *n* = 15), WT at 10°C (grey dots, *n* = 14), R185H at 20°C (red triangles, *n* = 15), R185H at 10°C (blue diamonds, *n* = 13). (**F**) Normalized peak current–voltage relationship curves for the WT at 20°C (black squares, *V*_1/2_ = −30.7 ± 1.3, *k* = 4.9 ± 0.4, *n* = 15), WT at 30°C (light blue stars, *V*_1/2_ = −34 ± 1, *k* = 5.2 ± 2.8, *n* = 12), R185H at 20°C (red triangles, *V*_1/2_ = −31.7 ± 1.7, *k* = 5 ± 0.5, *n* = 15), R185H at 30°C (pink asterisks, *V*_1/2_ = −33.6 ± 2, *k* = 4.8 ± 0.5, *n* = 10). Currents elicited from a holding potential of −100 mV to different test pulse potentials (50 ms) ranging from −80 to 40 mV in 5 mV increments. (**G**) Steady-state fast inactivation curves for the WT at 20°C (black squares, *V*_1/2_ = −85.8 ± 1.8, *k* = 7.5 ± 0.6, *n* = 14), WT at 30°C (light blue stars, *V*_1/2_ = −86.3 ± 2.1, *k* = 7.5 ± 0.3, *n* = 12), R185H at 20°C (red triangles, *V*_1/2_ = −86.9 ± 1.5, *k* = 7.6 ± 0.3, *n* = 14), R185H at 30°C (pink asterisks, *V*_1/2_ = −85.9 ± 2.4, *k* = 9 ± 1.5, *n* = 9). Currents elicited with test pulses to −10 mV following 500 ms inactivating prepulses. (**H**) Open-state fast inactivation kinetics for WT at 20°C (black squares, *n* = 15), WT at 30°C (light blue stars, *n* = 12), p.Arg185His at 20°C (red triangles, *n* = 15), R185H at 30°C (pink asterisks, *n* = 10). NFCI, non-freezing cold injury; NPD, neuropathic pain disorders; WT, wild type. Data are presented as mean ± SEM. Statistical analysis for group comparisons—two-way ANOVA with Sidak’s multiple comparison test (*statistically significant differences, *P* < 0.05).

Of the 40 participants recruited with non-freezing cold injury, six participants (7.5% allele frequency) carry the *SCN9A* p.Arg185His variant, which is associated with small fibre neuropathy.^12,36^ The six participants were all of the African descent (the majority from Ghana). Chronic non-freezing cold injury is an acquired painful sensory neuropathy observed almost exclusively in soldiers.^37^ Soldiers of African descent are disproportionately affected when compared to Caucasian soldiers. The allele frequency of p.Arg185His variant is <1% in gnomAD (1.4% in the African/African-American population); 165 individuals carried the allele in the general gnomAD population, 125 of which were Africans (166 allele counts, 1 was homozygote). Within NIHR Bioresource controls of African ancestry, p.Arg185His allele frequency was 0.0065 (2 out 104 participants,1.3%). In our cohort (Fig. 2A), the allele was significantly more common (6 out of the 38 participants) compared with both the general gnomAD population (Fisher’s Exact test, *P* = 1.4*10e−7, OR = 28.17, 95% CI [9.98, 65.45]), the gnomAD African population (Fisher’s Exact test, *P* = 0.001, OR = 5.66, 95% CI [2, 13.03]) and NIHR Bioresource African controls (Fisher’s Exact test, *P* = 7.6*10e−4, OR = 13.68, 95% CI [2.41, 143.26]).

The variant, p.Arg185His, results in an amino acid substitution in the linker between D1/S2 and D1/S3, which lies within a voltage sensing domain of Na_v_1.7 (Fig. 2B). This residue is highly conserved across all voltage-gated sodium channels. Functional studies have shown that p.Arg185His variant does not alter channel gating properties at room temperature; however, it was associated with enhanced resurgent currents and increases excitability when expressed in dorsal root ganglion neurons.^36^

Intronic variants and the detection of deletions were included in the gene-level analysis, and none were deemed medically actionable or of interest.

### The impact of *SCN9A* p.Arg185His variant on Na_V_1.7 channel function is temperature dependent

Non-freezing cold injury is caused by cold environmental exposure and neuropathic pain is worsened by further cold exposure. We hypothesized that there may be an interaction between Na_V_1.7 and an environmental trigger, such that cooling magnifies the effect of p.Arg185Hisvariant on channel function. WT and p.Arg185His Na_V_1.7 channels were expressed in combination with β1 and β2 subunits in HEK293T cells and Na_V_1.7 mediated currents recorded by whole-cell patch clamp (Fig. 2C–H). Changing the temperature from 20°C to 10°C did not significantly affect the half-activation potential of p.Arg185His (Fig. 2C), but significantly shifted the half-inactivation potential for steady-state fast inactivation of p.Arg185His mutant compared to WT (Fig. 2D). Channel kinetics were slower at 10°C compared with 20°C with similar effects for WT and p.Arg185His (Fig. 2E). We also wanted to assess channel behaviour at higher temperatures (30°C). Increasing the temperature from 20°C to 30°C did not affect *I*–*V* curve or the steady-state inactivation of the WT or the p.Arg185His (Fig. 2F and G). Faster inactivation kinetics were observed at 30°C and the change was similar for WT and p.Arg185His (Fig. 2H).

In conclusion, the p.Arg185His mutant exhibited a depolarizing shift of half-inactivation potentials at 10°C but not at 20°C or 30°C, which is a change in channel gating consistent with a gain of function only at lower temperatures.

### Gene-wise rare variant association testing results

When comparing European participants against controls, gene-wise, rare variant association testing for Tiers 1–3 genes identified six genes and regulatory regions of four genes with a significant difference in rare variant distribution (Table 7). In total, 177 018 genomic loci were called in Tier 1–3 genes, 132 605 SNPs (93 295 were singletons), 4059 insertions and 8498 deletions (8859 out of the 12 557 were singletons). Variants per loci rate was 0.000046 in priority genes regions. Heterozygosity to Homozygosity ratio was 0.96, 1.35 for SNPs and 0.29 for INDELS. For European participants, gene-wise rare variant association varied according to clinical phenotype (Supplementary Table 2), and 20 genes and 24 regulatory regions reached Bonferroni-adjusted significance for at least 1 phenotype. For African participants, several genes showed similar associations with post-traumatic neuropathy, small fibre neuropathy and non-freezing cold injury (Supplementary Table 2).

**Table 7:** Results from the gene-wise association test (using the gene-wise approach of the combined burden and variance-component test SKAT-O) for rare variants of Tier 1–3 genes and functionally validated variants of the *SCN9A, SCN10A, SCN11A* genes for all participants with neuropathic pain

Genomic coordinates and the HGNC gene symbol for gene models	Fraction with rare variants	Number of variants considered	*P*-value	Rho
**Europeans**				
**Tier 1 pain genes**				
All neuropathic pain (*n* = 126) versus controls (*n* = 5096)				
1:115828713-115836247_NGF	0.0026346	14	2.053e−08	0
3:38387651-39150434_SCN11A_promoter	0.0037214	186	1.7809e−06	0
**Tier 2–3 pain genes**				
All neuropathic pain versus controls				
1:212733740-213037331_FLVCR1_promoter	0.07838	704	1.0404e−05	0
2:241653459-241759532_KIF1A	0.038202	180	8.74e−08	0
2:241757820-241808205_KIF1A_promoter	0.011691	182	1.5858e−06	0
12:52056606-52201160_SCN8A	0.0097151	44	5.0888e−08	0
2:234835229-234916724_TRPM8	0.017948	90	2.9788e−06	0
**Tier 1-3 pain genes**				
Probable/definite neuropathic pain versus controls				
1:212733740-213037331_FLVCR1_promoter	0.078509	704	2.0274e−07	0
2:241656788-241737150_KIF1A	0.038265	180	2.7235e−09	0
2:241757820-241808205_KIF1A_promoter	0.01171	182	2.4975e−07	0
1:115828713-115836247_NGF	0.002639	14	2.6429e−09	0
12:52056606-52201160_SCN8A	0.0097312	44	1.5018e−09	0
2:167227721-167351474_SCN9A_promoter	0.037605	461	2.3805e−05	0
8:72935185-72987631_TRPA1	0.015009	73	5.2482e−05	0
2:234835229-234916724_TRPM8	0.017978	90	2.825e−07	0
*SCN9A, SCN10A, SCN11A* functionally validated variants				
3:38739727-38793804_SCN10A (Small fibre neuropathy)	0.0013329	2	0.00016219	0

Significance was set to the Bonferroni-adjusted 0.01 threshold for the number of genes considered (0.01/36 = 2.7*10e−4). We report unadjusted *P*-values that passed the Bonferroni corrected significant threshold. The genomic coordinates and the HGNC gene symbol of the gene models that have a significantly different configuration of rare variants between cases and controls are presented in the first column. The second column holds the fraction of individuals carrying rare variants below the MAF threshold < 0.05. We show the number of variants considered for the association, followed by the *P*-value of the association test. The last column holds the Rho statistic. Values can be interpreted as Rho = 0, causal and non-causal variants with opposing directions driving the association to Rho = 1, high percentage of causal variants acting in the same direction driving the association. Results are grouped by ethnicity and phenotype.

For the groups of functionally validated voltage-gated sodium channel variants, a significant association of *SCN10A* with small fibre neuropathy in Europeans was found. This was driven by two gain-of-function variants, *SCN10A(ENST00000449082.2):c.4984G>A, p.Gly1662S* ((Allele frequency 0.0039 in Neuropathic Pain and 0.0005 in controls) and *ENST00000449082.2:c.1661T>C, p.Leu554Pro* (Allele frequency 0 in Neuropathic Pain and 0.00008 in controls) (Table 7). These variants are reported in patients with small fibre neuropathy, cause a gain of function of the Na_V_1.8 channel and enhance dorsal root ganglion neuron excitability.^38,39^ However, both variants were characterized as likely benign in ClinVar and their allele frequencies in gnomAD suggest that they cannot be the only cause of neuropathic pain. No significant associations were found for *SCN9A*, nor *SCN11A*.

For the whole gene set analysis, 146 genes reached Bonferroni corrected significance in Europeans with neuropathic pain when compared to controls (Supplementary Table 3). Four of the genes, *KIF1A*, *KCNQ5, KCNK4* and *NOS2* are linked to human neuropathic pain.

### The *TRPA1* p.Ala172Val variant is associated with episodic pain and gain of channel function

**Figure 3:**
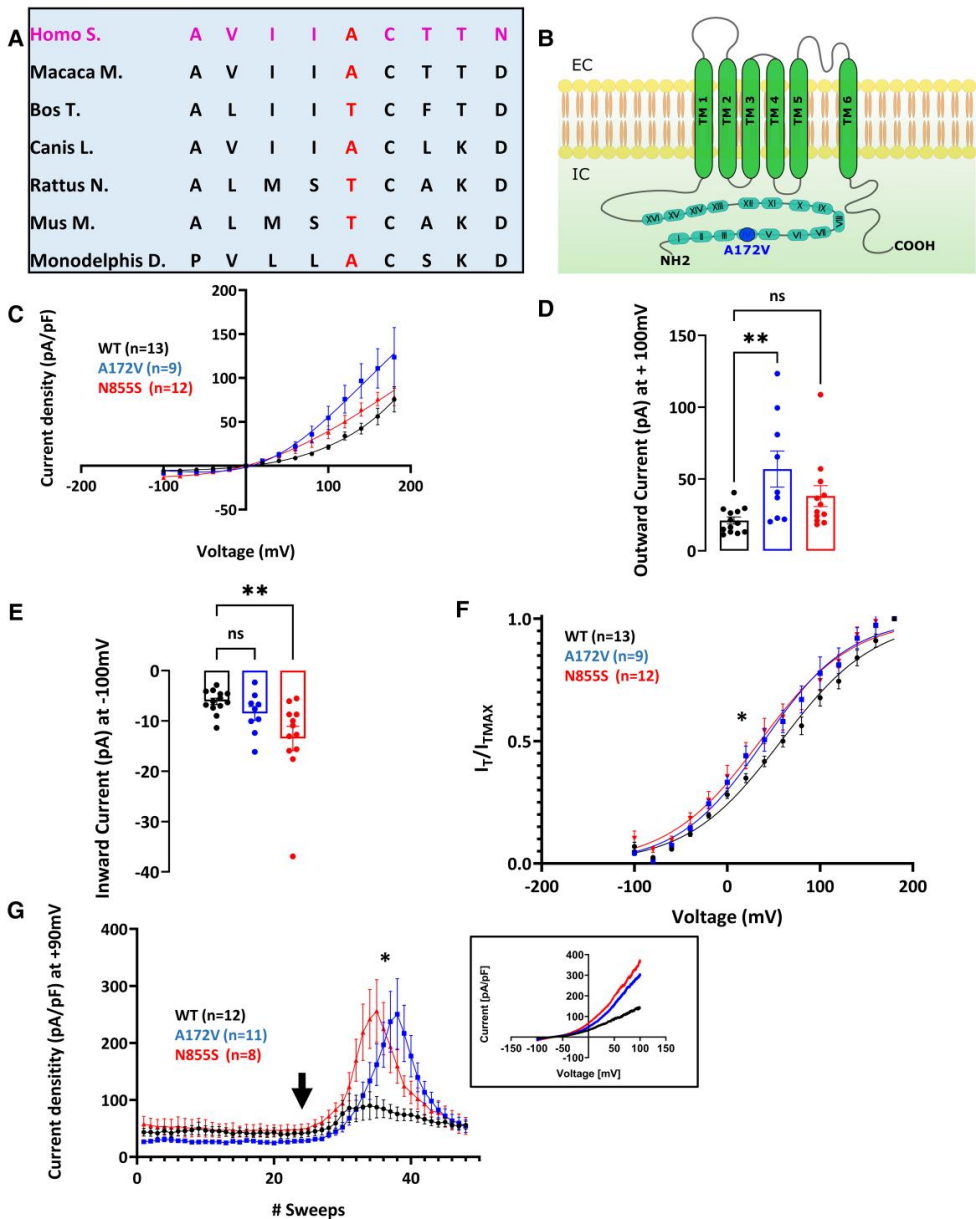
**Description and biophysical characterization of hTRPA1 p.Ala172Val variant, which shows gain of function in response to AITC.** (**A**) p.Ala172Val variant: Alanine is substituted with a valine in the fourth ankyrin repeat domain of the channel. The residue is moderately conserved across different mammalian species. (**B**) Schematic of TRPA1 channel topology. p.Ala172Val variant is represented with a blue dot. Voltage-dependence activation of hTRPA1 p.Ala172Val and p.Asn855Ser was measured in response to AITC. Current–voltage curves were measured with a two-voltage-step protocol (voltage ramps ranging from −100 to + 100 mV for 500 ms, every 5 s). Holding potential was set at −70 mV. Application of 25 µM of AITC shows enhanced activity of p.Ala172Val variant in response to 25 μM AITC in the presence of extracellular calcium alone (**C–G**). (**C**) Current density, assessed using a two-voltage-step protocol, was significantly different between WT and variant channels as quantified in (**D**) and (**E**). (**D**) Outward currents (+100 mV, WT = 21.12 ± 2.46 pA/pF, p.Arg185His = 56.92 ± 12.52 pA/pF, p.Asn855Ser = 38.12 ± 10.77 pA/pF) were significantly increased for p.Ala172Val (*P* < 0.005). (**E**) Inward currents (−100 mV, WT = −6.12 ± 0.63 pA/pF, p.Arg185His = −8.48 ± 1.37 pA/pF, p.Asn855Ser = −13.48 ± 2.40 pA/pF) were significantly increased for p.N885S (*P* < 0.005). (**F**) Tail current analysis showed a significant shift in half-maximal activation potential for both p.Ala172Val (*n* = 9, *V*_1/2_ = 35.55 ± 5.45 mV, and p.Asn855Ser, *V*_1/2_ = 37.03 ± 8.28 mV, *n* = 12, when compared with WT *n* = 13, *V*_1/2_ = 59.16 ± 5.25 mV; *P* < 0.05). Slopes of the voltage-activationcurve (i.e. voltage sensitivity) (*k* = 49.38 ± 2.03 mV WT, *k* = 43.29 ± 2.63 mV A172V, *k* = 45.20 ± 2.36 mV; *P* = 0.18) were not significantly different. (**G**) Current–voltage relationships were tested with a voltage-ramp protocol in which voltage changes at a steady rate and the resulting current is recorded. After administration of 25μM (arrow) averaged currents from voltage ramps were extrapolated and at +90 mV showed a 3-fold increase in current densities at positive potentials after the application of 25 µM AITC (WT = 85.13 ± 23.30 mV, p.Ala172Val = 250.4 ± 62.39 mV, p.Asn855Ser = 256.1 ± 54.90 mV; *P* < 0.05). Insert shows example tracing. WT, wild type. Data are presented as mean ± SEM. Statistical analysis for group comparisons—one-way ANOVA with Sidak’s multiple comparison test (*statistically significant differences, *P* < 0.05).

Variants in Tier 2 and 3 genes were filtered (Tables 3–5), using the same approach as for Tier 1 genes to identify those that may be pathogenic. A rare TRPA1 variant, p.Ala172Val (gnomAD allele frequency 0.0003), was identified in a participant who suffers from episodic widespread chronic pain with neuropathic characteristics particularly affecting the trunk. Clinical phenotype was similar to a neuropathic pain syndrome associated with a TRPA1 channelopathy (p.Asn855Ser variant)^32^; however, the participant’s pain was not precipitated by physiological stressors. MRI of the brain and spine, nerve conduction studies and skin biopsy of the lower leg were within age- and gender-appropriate reference ranges. The participant’s child, carrying the same variant, was similarly affected by chronic abdominal and pelvic pain with normal investigations. *In silico* tools, Polyphen and SIFT scores, predicted p.Ala172Val to be deleterious. The variant is moderately conserved across species (Fig. 3A), is situated within the ankyrin repeat domain of the protein (Fig. 3B) and is a non-polar to non-polar amino acid exchange (Grantham score 64). Based on the rarity of the variant, clinical phenotype consistent with the TRPA1 Familial Episodic Pain Syndrome, positive family history and *in silico* analysis showing the variant to be in the poorly understood N-terminal ankyrin repeats, the *TRPA1* p.Ala172Val variant was prioritized for functional studies. These were compared to the *TRPA1* variant p.Asn855Ser which is the only variant previously linked to this disorder.

**Table 3:** A summary of the genes classified as tier 2 genes

Gene symbol	HGNC database: ID	Chromosomal position	HGNC database: description	Clinical phenotypes	OMIM	PMID reference
**Tier 2 genes**						
*ATL1*	HGNC:11231	14q21.3	Atlastin GTPase 1	HSAN 1; Hereditary Spastic Paraplegia	613708	21194679, 22340599
*ATL3*	HGNC:24526	11q13.1	Atlastin GTPase 3	HSAN 1	615632	24459106, 30680846
*CCT5*	HGNC:1618	5p15.2	Chaperonin containing TCP1 subunit 5	HSAN with spastic paraplegia	256840	16399879
*CLTCL1*	HGNC:2093	22q11.2	Clathrin heavy chain like 1	HSAN 5/congenital insensitivity to pain	601273	26068709
*KIF1A*	HGNC:888	2q37.2	Kinesin family member 1A	HSAN 2; Hereditary Spastic Paraplegia	614213	21820098, 25265257
*MPV17*	HGNC:7224	2p23.3	MPV17, mitochondrial inner membrane protein	Congenital neuropathy leads to absent pain at birth in severe cases. Hepatic failure and encephalopathy overshadow the neuropathy	256810	185990, 11431741
*NAGLU*	HGNC:7632	17q21.2	*N*-acetyl-alpha-glucosaminidase	Painful axonal polyneuropathy in heterozygotes; mucopolysaccharidosis IIIB when homozygous		25818867
*TRPA1*	HGNC:497	8q13	Transient receptor potential cation channel subfamily A member 1	Familial episodic pain syndrome	615040	20547126,16564016

If genes were implicated in neuropathic pain, but did not meet Tier 1 criteria (see Table 2), they were classified as Tier 2 genes.

HSAN, Hereditary and Autonomic Sensory Neuropathy; HGNC, HUGO Gene Nomenclature Committee.

The biophysical properties of WT, p.Ala172Val and p.Asn855Ser hTRPA1 were compared (Supplementary Fig. 3). In HEK293T transfected cells, TRPA1 current–voltage relationship and half-maximal activation for WT, p.Ala172Val and p.Asn855Ser were not statistically different. Under control conditions, WT, p.Ala172Val and p.Asn855Ser current traces showed sustained outward rectification at positive potentials, consistent with an underlying voltage dependence of channel gating. As current density did not differ between WT and the variants, under control conditions, it is unlikely that channel trafficking is affected in p.Ala172Val and p.Asn855Ser channels.

Voltage-dependence activation of *hTRPA1* p.Ala172Val and p.Asn855Ser was measured in response to mustard oil (AITC, a TRPA1 electrophilic agonist) and Menthol (non-electrophilic agonist of TRPA1). In response to 25 µM AITC, in the presence of extracellular calcium, p.Ala172Val showed a pronounced linearization of the current–voltage relationship compared to WT in response to a two-voltage-step protocol. A steeper activation curve was observed at positive potentials for both p.Ala172Val and p.Asn855Ser compared with WT (Fig. 3C). Currents were significantly increased at positive potentials for p.Ala172Val (Fig. 3D) and at negative potentials for p.Asn855Ser (Fig. 3E). Analysis of tail currents demonstrated a significant leftward shift of voltage dependence of channel activation in the presence of AITC for both variants (Fig. 3F); however, the slope of the voltage-activation curve did not significantly change. Current–voltage relationships, tested with a voltage-ramp protocol, showed an increase in current densities at positive potentials after the application of 25 µM AITC (Fig. 3G). In summary, these findings show an enhanced response of p.Ala172Val to 25 µM AITC, suggesting a gain-of-function behaviour of this variant, under agonist stimulation.

**Figure 4:**
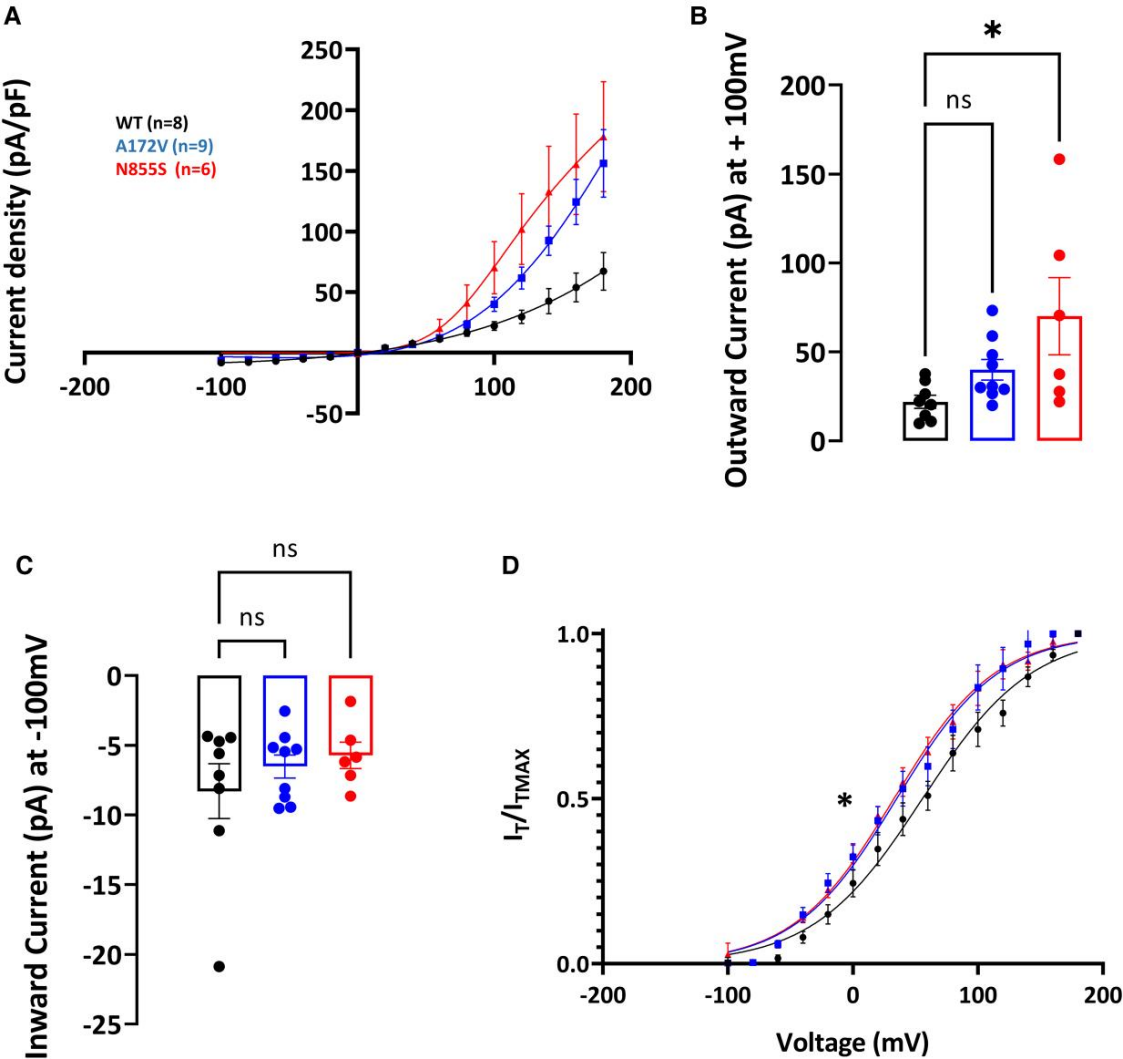
**hTRPA1p.Ala172Val variant shows enhanced activity after the application of 100 µm of menthol in the presence of intracellular and extracellular calcium.** Current–voltage curves were measured with a two-voltage-step protocol (voltage ramps ranging from −100 to + 100 mV for 500 ms, every 5 s; holding potential was set at −70 mV). (**A**) Current density, assessed using a two-voltage-step protocol, in WT and variant channels were significantly different as quantified in (**D**) and (**E**). (**B**) Outward currents were enhanced for both p.Ala172Val and p.Asn855Ser (+100 mV, WT = 21.96 ± 3.64 mV, p.Ala172Val = 39.94 ± 5.80 mV, and p.Asn855Ser = 70.07 ± 21.71 mV; *P* < 0.05). (**C**) Inward currents were not statistically different (−100 mV, WT = −8.29 ± 1.97 mV, −6.52 ± 0.83 mV p.Ala712Val, and p.Asn855Ser = −5.72 ± 0.94 mV; *P* = 0.58, and *P* = 0.40, respectively). (**D**) Half-maximal activation potential was significantly shifted leftward for p.Ala172Val and p.Asn855Ser (V1/2, WT = 61.83 ± 7.37 mV *n* = 8, p.Ala172Val = 36.41 ± 7.58 mV *n* = 9, p.Asn855Ser = 33.60 ± 6.67 mV *n* = 6). Voltage sensitivity was significantly altered (slope factor *k* = 41.51 ± 2.33 mV WT; 40.21 ± 2.98 mV p.Arg185His; 39.32 ± 3.03 mV p.Asn855Ser; *P* < 0.05). WT, wild type. Data are presented as mean ± SEM. Statistical analysis for group comparisons—one-way ANOVA with Sidak’s multiple comparison test (*statistically significant differences, *P* < 0.05).

Application of 100 µM Menthol did not significantly change current density nor voltage sensitivity when applied to p.Ala172Val or p.Asn855Ser in the presence of extracellular calcium (Supplementary Fig. 4). However, in the presence of both extracellular and intracellular calcium, significant changes were observed (Fig. 4A–D). Both p.Ala172Val and p.Asn855Ser channels showed an increase in outward currents and voltage sensitivity in response to 100 µM Menthol.

In summary, the p.Ala172Val variant confers gain-of-function properties on TRPA1 channel in response to the agonists AITC and Menthol. For the latter, the effect was dependent on intracellular calcium.

## Discussion

In this study, we applied WGS to a cohort of patients with neuropathic pain disorders. We carried out two analyses. First, we identified medically actionable variants and second, we carried out group-wise association tests at the gene level for rare variants. The diverse phenotypes ranged from congenital insensitivity to pain to painful neuropathy. Clinically relevant findings in genes associated with pain were reported in 12% of participants. The majority of clinically relevant variants were in voltage-gated sodium channels. We made new genotype–phenotype associations, such as the Na_V_1.7 p.Arg185His variant which was more frequent in Africans with non-freezing cold injury. We provide new mechanistic insights showing that p.Arg185His interacts with cold, causing a gain of function in the Na_V_1.7 gating properties. The gain of function of p.Ala172Val in TRPA1 in response to agonists extends our knowledge of painful TRPA1 channelopathies.

The study of genetic neuropathic pain disorders poses challenges in describing clinical phenotype and assigning pathogenicity to associated variants. We used the gold standard grading system, of the Neuropathic Pain Special Interest Group of IASP.^27^ Such an approach works well in disorders where there is structural injury to sensory neurons. For example, in painful distal symmetrical polyneuropathy, pain and sensory signs are found in a neuroanatomically plausible distribution (meeting probable criteria), and specialized investigations confirm a lesion of the somatosensory nervous system (meeting definite criteria). However, the majority of Mendelian pain disorders are sensory neuron ion channelopathies, in which pain is episodic, with no structural injury to sensory neurons. For example, in Inherited Erythromelalgia (Na_V_1.7) or Familial Episodic Pain Syndrome (TRPA1, Na_V_1.9), sensory examination (between pain episodes), neurophysiology and cutaneous innervation are normal. Careful attention to clinical history is, therefore, essential.

We identified medically actionable variants in 12% of the participants. In our cohort, five participants carry pathogenic or likely pathogenic variants. Four pathogenic variants were in voltage-gated sodium channels, which can impact patient care. For example, a pair of sisters with Inherited Erythromelalgia carry the pathogenic *SCN9A* p.Ile848Thrwith autosomal dominant pattern of inheritance. Only a minority of patients with Inherited Erythromelalgia possess *SCN9A* mutations.^40^ Identification of a genetic cause for erythromelalgia means family genetic counselling and preferential treatment with non-selective sodium channel blockers,^41^ which is not the standard treatment for other causes of neuropathic pain.^42^ The family may also access future treatments such as selective sodium channel blockers.^43^

A further 20 participants carry VUS in voltage-gated sodium channels that were deemed clinically relevant. Ascribing pathogenicity to ion channel variants is difficult. The majority are relatively common, exhibit subtle channel gain-of-function effects and likely interact with environmental factors. For example, in our cohort the *SCN9A(ENST00000409672.1)*: c.2215A>G, p.Ile739Valvariant was found in four participants with painful neuropathy and described previously as pathogenic.^36^ However, p.Ile739Valis common in the general population (0.2% allele frequency), while the prevalence of small fibre neuropathy is rare (∼50 per 100 000^44^). It is unlikely that this variant is fully penetrant (thus pathogenic); but may act as a risk factor. Nevertheless, it is important to identify such variants for treatment selection, because Lacosamide (a sodium channel blocker) has shown efficacy in a clinical trial of patients with small fibre neuropathy and rare *SCN9A*^45^ variants. Voltage clamp analyses can add valuable experimental evidence to the pathogenicity assessment that cannot be replaced by *in silico* prediction tools, as *in silico* analysis is not comprehensive and variant’s effect prediction software are likely to underestimate the impact of the gain-of-function effects. However, recent advances in ion channel modelling have improved outcomes.^46^

WGS is now integrated into the UK national health system (see https://www.genomicsengland.co.uk/) and is now considered the standard of care across the UK.^47^ As of 2022 all testing at NHS genomic centres for painful neuropathies and channelopathies is via WGS. The panel application ‘Inherited neuropathies or pain disorder v1.36’ was informed by the NIHR Bioresource (100 000 genomes), our functional studies and includes all the tier 1 pain genes in this paper and *TRPA1*. Such an approach will identify many more new variants and the findings of our study will be of direct relevance to practitioners assessing patients with neuropathic pain. Understanding how these novel variants relate to clinical phenotype presents significant challenges for clinicians. The combined expertise of our multidisciplinary team meetings was vital in the interpretation of variant pathogenicity. We note that the proportion of cases in which we have found a pathogenic variant that explains the clinical pain phenotype was low (2.4%), although in a higher proportion (a further 10%) the finding was clinically actionable. In many cases, this was VUS in which the relationship to the clinical phenotype could not be causally established, but could still have implications for treatment and so was reported. The low yield in solving cases at a diagnostic level partly represents the fact that neuropathic pain is monogenic in only a minority of cases. In many cases it is likely to arise from gene–environment interaction and/or multiple genes. To try and improve diagnostic yield in the future we could enrich analysis pipelines (for instance to include analysis of repeat expansions in the WGS data), expand co-segregation analysis and also functional analysis. It is not possible to test all novel variants with current patch clamp technology. Automated prediction of pathogenicity and higher throughput functional assays should be a priority, and important next steps in the integration of WGS into healthcare.

We were able to extend genotype–phenotype associations of *SCN9A*. The *SCN9A* p.Arg185His variant was more common in study participants with non-freezing cold injury. p.Arg185His is associated with neuropathic pain disorders, such as small fibre neuropathy^12^ and painful diabetic neuropathy.^13^ Non-freezing cold injury is a chronic neuropathic pain disorder caused by an acquired sensory neuropathy^37^ after prolonged exposure to cold and wet environments. Further cold exposure intensifies the neuropathic pain in a similar manner to cold allodynia after platinum-based chemotherapy.^48^ The mechanism for this cold allodynia is unknown. We show that p.Arg185His displays gain-of-function characteristics at lower temperatures. This occurs through a shift of fast inactivation at 10°C, but not at 30°C or room temperature (20°C). Pathogenic *SCN9A* variants that cause inherited erythromelalgia demonstrate temperature sensitivity at biophysical and clinical levels such that warming intensifies pain and cooling is analgesic.^49^ A link, therefore, exists between the biophysical properties of Na_V_1.7 channel, clinical phenotype and temperature sensitivity. An increase of p.Arg185His variant excitability on cooling may contribute to cold allodynia in a sub-group of non-freezing cold injury patients. Another consideration is that increased activity of p.Arg185His at cold temperatures could injure nerves through calcium dependant excitotoxicity.^50,51^ In summary, p.Arg185His may contribute to clinical phenotype or increase the risk for cold-induced neuropathic pain, but we cannot conclude that is causative in isolation.

Bevimed is an inference procedure for identifying loci associated with rare hereditary disorders using Bayesian model comparison and was used in an analysis of all cohorts of NIHR BioResource Rare Diseases.^19^ Under different modes of inheritance there was strong evidence for 95 genes and 29 binary disease tags (cases versus controls). No diseases from our cohort were among the associations with strong evidence. We used SKAT-O in our cohort. Group-wise association tests can increase the power to detect associations between rare alleles and phenotypes by aggregating variant counts on the gene level that can have an impact on gene function. Neuropathic pain can arise as a complex effect of rare variants with causal effects in the same direction, which the burden test is best powered to detect, or effects with different levels of causality and direction, which the sequence kernel association test is best powered to detect. We used an approach that maximizes test power by considering the combination of both the burden and sequence kernel association tests. In testing for associations for the whole gene set in Europeans with neuropathic pain, 146 genes reached statistical significance. These are gene-wise results showing a significant association of a configuration of rare alleles grouped at the gene level with certain neuropathic pain phenotypes. In most of the associations, the variant-component test was better powered. This is indicated by the low Rho statistics empirically calculated from the data and is suggestive of the presence of variants with different values of causality and opposing direction of effects towards the phenotype, i.e. both protective and deleterious variants present and driving the association. We focused on bi-allelic variants and indels of high quality, with moderate to high impact on protein-coding genes, as we had a moderate sample size and did not want to diminish statistical power after Bonferroni correction. The genes *KCNK4*, *KCNQ5* and *NOS2* were significantly associated with neuropathic pain after gene-wise rare variant association testing. These have not been previously linked to human pain at a genetic level, but preclinical models have implicated these genes in pain pathogenesis.^52-54^*KIF1A*, a Tier 2-3 priority gene also reached genome-wide significance. The association with neuropathic pain would require replication in independent cohorts and functional studies.

We undertook an analysis focusing on genes known or likely to be implicated in neuropathic pain, and we, therefore, used more relaxed criteria in filtering rare variants, including modifier, moderate and high-impact variants. We also note that our *in silico* variant effect predictions are limited by their tendency to underestimate gain-of-function effects. Our analysis showed an association of five Tier 2 genes, including *TRPA1*, with neuropathic pain. TRPA1 is a calcium-permeable non-selective cation channel that acts as a sensor of noxious external stimuli, such as mustard oil (AITC) and Menthol. The p.Asn855Ser TRPA1 variant is associated with familial episodic pain syndrome characterized by truncal pain triggered by physiological stress or exercise.^32^ We identified a study participant with a similar clinical phenotype of truncal pain who carried the p.Ala172Val TRPA1 variant. p.Ala172Val is a missense mutation in the fourth domain of the Ankyrin region. Our *in vitro* electrophysiological studies show that TRPA1 p.Ala172Val variant causes a gain of function in response to agonist stimulation and provides evidence for Ca^2+^-mediated channel activation through the Ankyrin repeat domains of TRPA1 channel.

TRPA1 consists of a large intracellular NH2 and COOH termini, with the NH2 terminus containing an elongated ankyrin repeat domain which is highly conserved. Human TRPA1 consists of 16 ankyrin repeat domains that connect to the transmembrane domains *via* a linker region. The p.Ala172Val variant did not change the biophysical properties of TRPA1 channel in the naïve state, but did enhance responses to AITC and Menthol. Current density was larger for AITC when compared with Menthol. Due to differences in their electrophilic nature, agonists vary in their ability to covalently (AITC) or noncovalently (Menthol) modify the channel upon binding. This difference in channel binding might underlie different agonist responses. The p.Asn855Ser and p.Ala172Val variants increase activation in response to agonists, although with distinct impacts on the biophysical properties of the channel. Intrinsic differences due to the positions of the variants within the channel (p.Asn855Ser is in the transmembrane domain S4), might underlie the differences observed. Our data show that the ankyrin repeat domain is important in TRPA1 channel activation and gating functions. The p.Ala172Val variant is reported at a heterozygous frequency of 0.0003 in gnomAD and individuals over 70 years of age are reportedly healthy so it is unlikely to be fully penetrant. It is more likely to act as a risk factor and contribute to the expression of neuropathic pain by altering the functional properties of nociceptive afferents; however, the drivers for channel activation (environmental versus endogenous ligands) are not known.

In our study, we identified clinically relevant variants in 12% of the participants, with an impact on clinical care and treatment. The majority of these variants are located in ion channels which are enriched in nociceptors and environmental triggers (such as cold in the case of non-freezing cold injury) may interact and enhance the gain-of-function impact of such variants.

## Supplementary Material

fcad037_Supplementary_Data

## Data Availability

The genotype and phenotype data can be accessed by application to the NIHR BioResource Data Access Committee at dac@bioresource.nihr.ac.uk or by application to Genomics England Limited following the procedure outlined at https://www.genomicsengland.co.uk/research.

## Abbreviations

AITC =: Allyl isothiocyanate
HGNC =: HUGO Gene Nomenclature Committee
HSAN =: Hereditary and Autonomic Sensory Neuropathy
MAF =: Minor allele frequency
NFCI =: Non-freezing cold injury
NIHR =: National Institute for Health and Care Research
NOS =: Not otherwise specified
NPD =: neuropathic pain disorders
SNP =: single nucleotide polymorphism
VUS =: variant of uncertain significance
WT =: Wild type
