# Supplementary material for: Investigating genotype–phenotype relationship of extreme neuropathic pain disorders in a UK national cohort
Source: Brain Commun. 2023 Feb 20;5(2):fcad037. doi: 10.1093/braincomms/fcad037 (PMC9991512; doi:10.1093/braincomms/fcad037)
Supplement: fcad037_Supplementary_Data [file fcad037_supplementary_data.pdf]

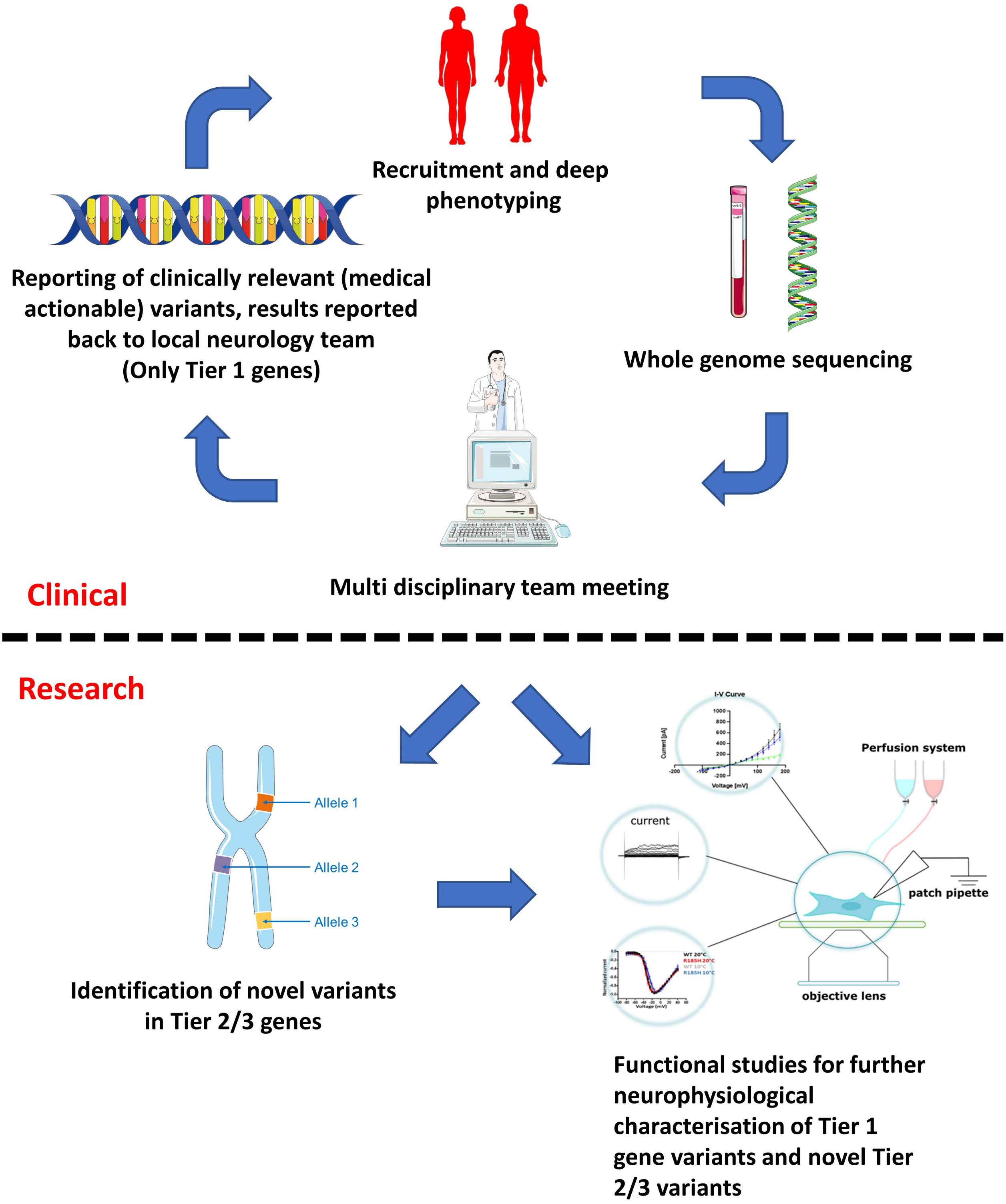

Recruitment and deep phenotyping
Whole genome sequencing
Multi disciplinary team meeting
Reporting of clinically relevant (medical actionable) variants, results reported back to local neurology team
(Only Tier 1 genes)
Clinical
Research
Allele 1
Allele 2
Allele 3
Identification of novel variants in Tier 2/3 genes
I-V Curve
Current [pA]
Voltage [mV]
current
Perfusion system
patch pipette
objective lens
Functional studies for further neurophysiological characterisation of Tier 1 gene variants and novel Tier 2/3 variants

**Supplementary figure 1**
**Flow diagram outlining steps in the project.**

The flow diagram outlines recruitment of participants, analysis pipeline, genetic testing and the difference between the clinical reporting of relevant gene variants versus the exploratory gene analysis and in vitro functional assessment of variants of interest

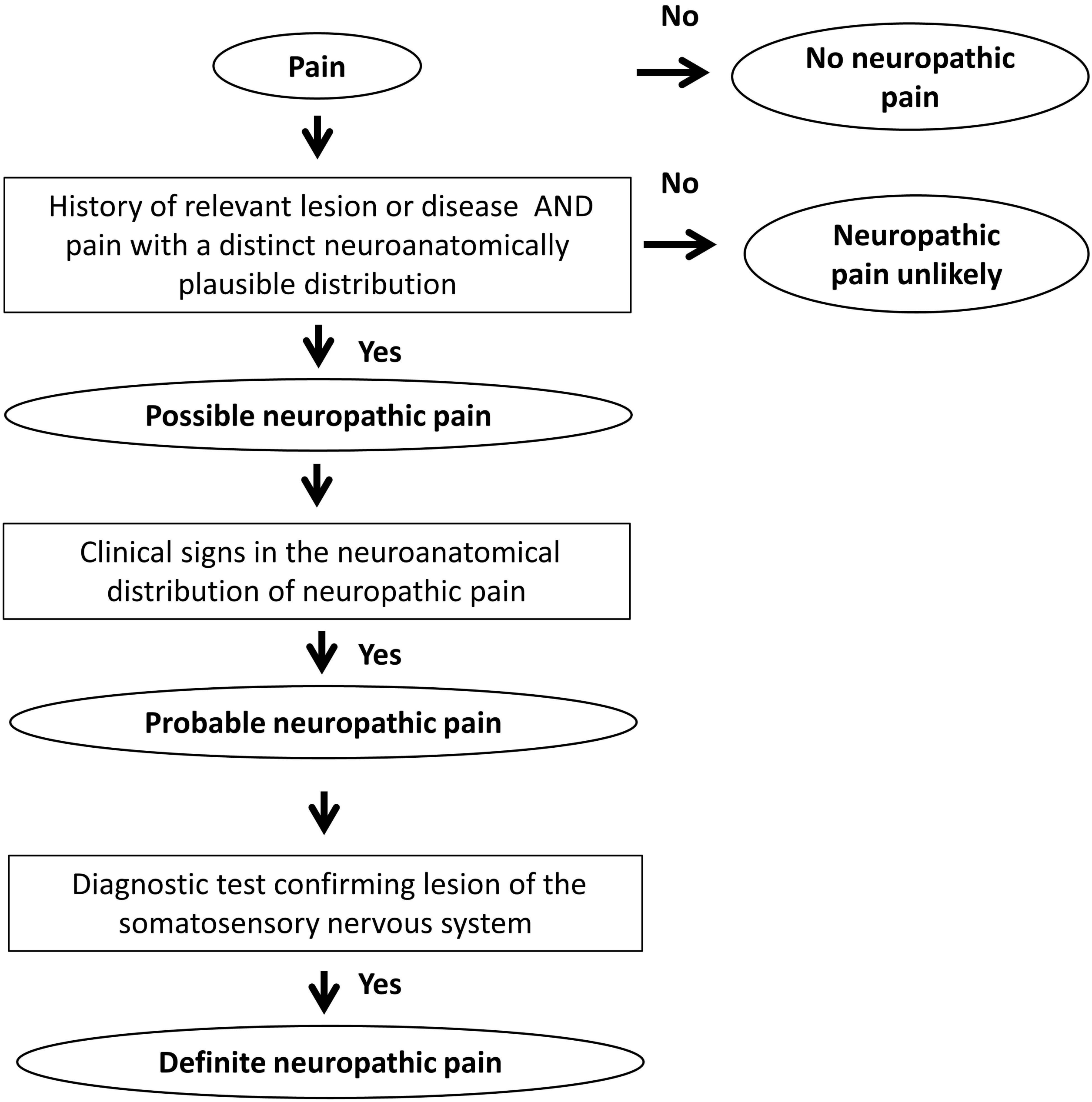
Pain
No
No neuropathic pain
History of relevant lesion or disease AND pain with a distinct neuroanatomically plausible distribution
No
Neuropathic pain unlikely
Yes
Possible neuropathic pain
Clinical signs in the neuroanatomical distribution of neuropathic pain
Yes
Probable neuropathic pain
Diagnostic test confirming lesion of the somatosensory nervous system
Yes
Definite neuropathic pain

**Supplementary figure 2**
**Flow diagram outlining neuropathic pain grading .**

Possible neuropathic pain must fulfil criteria 1 and 2.
Probable neuropathic pain must fulfil criteria 1, 2 and 3.
Definite neuropathic pain must fulfil all 4 criteria.

1. Pain with a distinct neuroanatomically plausible distribution e.g. pain symmetrically distributed in the extremities, pain in erythematous areas
2. A history suggestive of a relevant lesion or disease affecting the peripheral or central somatosensory system e.g. a history of neuropathy symptoms including decreased sensation, or positive sensory symptoms e.g. burning, aching pain mainly in the toes, feet or legs.
3. Demonstration of distinct neuroanatomically plausible distribution of neuropathic pain e.g. presence of clinical signs of peripheral neuropathy such as decreased distal sensation or decreased/absent ankle reflexes.
4. Demonstration of the relevant lesion or disease by at least one confirmatory test e.g. abnormality on either nerve conduction tests, thermal thresholds or intra-epidermal nerve fibre density.

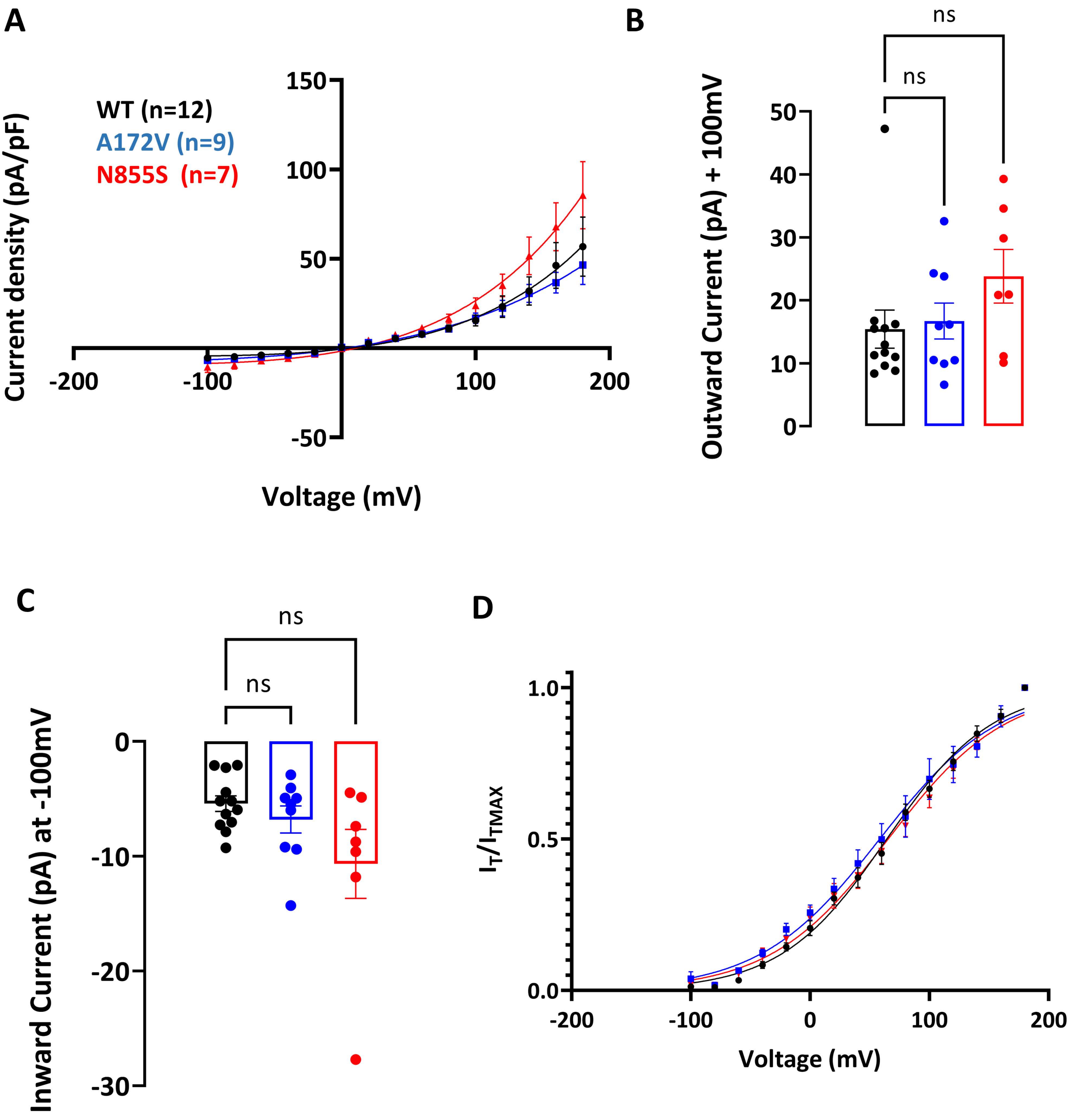
A
WT (n=12)
A172V (n=9)
N855S (n=7)
150
100
50
-50
-200
-100
100
200
Current density (pA/pF)
Voltage (mV)
B
ns
ns
Outward Current (pA) + 100mV
50
40
30
20
10
0
C
ns
ns
Inward Current (pA) at -100mV
0
-10
-20
-30
D
$I_T/I_{TMAX}$
1.0
0.5
0.0
-200
-100
0
100
200
Voltage (mV)

**Supplementary figure 3**
**Biophysical characterisation of hTRPA1 WT, p.Ala172Val and p.Asn855Ser variant in control conditions shows no significant differences.**

Voltage step protocol consisted of 400 ms voltage steps to test potentials ranging from –100 mV to +180 mV, followed by a final invariant step to -75 mV (400 ms to measure tail currents). The holding potential was set at -0 mV.
A) Current density measured in WT and mutant channels were not statistically different as quantified in b) and c)
B) Outward currents at (+100mV, WT = 15.41 ± 3.01pA/pF, p.Ala172Val = 16.69 ± 2.85pA/pF, p. Asn855Ser = 23.81 ± 4.25pA/pF ) were not significantly different between WT and the respective variants (p = 0.77 WT versus p.Ala172Val, p = 0.17 WT versus p.Asn855Ser)
C) Inward currents (at -100mV, WT = -5.42 ± 0.68pA/pF, p.Ala172Val = -6.80 ± 1.18pA/pF and p.Asn855Ser = -10.66 ± 3.00pA/pF), were not significantly different between WT and the respective variants (p = 0.75 WT versus p.Ala172Val, and p = 0.05 WT versus p.Asn855Ser).
D) Voltage of half-maximal activation for WT n = 12, p.Ala172Val n = 9 and p.Asn855Ser n = 7 ($V_{1/2}$, WT = 59.70 ± 4.59mV, p.Ala172Val = 59.45 ± 8.91mV, p.Asn855Ser = 64.85 ± 8.09mV; p= 0.99, and p = 0.85, respectively) and slopes of the voltage-activation curve (k, WT = 42.51 ± 2.01mV, p.Ala172Val = 46.19 ± 2.60mV, p.Asn855Ser = 47.45 ± 1.88mV; p = 0.41 and p = 0.26, respectively) were not statistically different.

WT – Wild Type
Data are presented as mean ± SEM.
Statistical analysis for group comparisons - One way ANOVA with Sidak's multiple comparison test (* - indicates statistically significant differences, P <0.05)

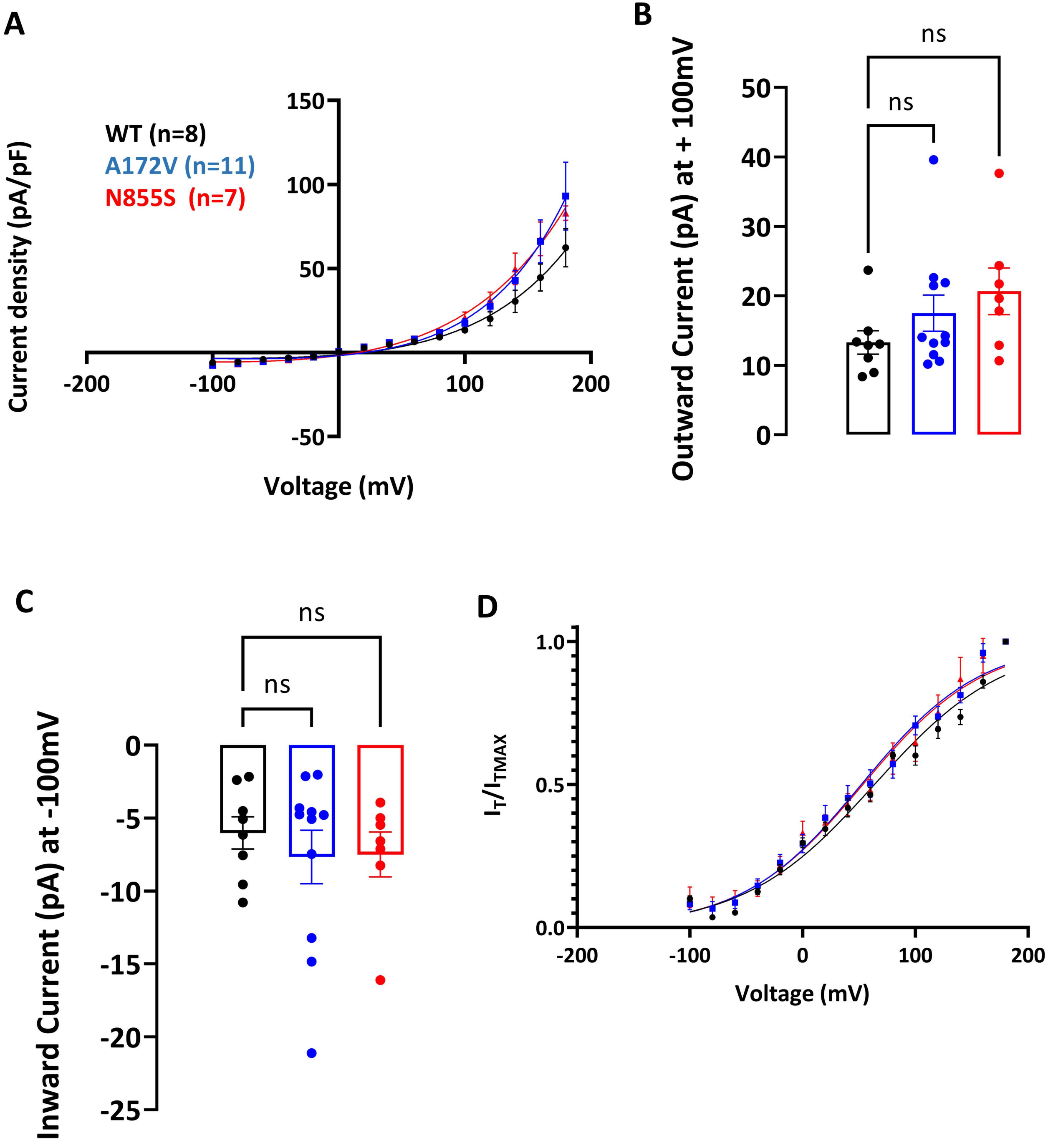
A
WT (n=8)
A172V (n=11)
N855S (n=7)
Current density (pA/pF)
150
100
50
-50
-200
-100
100
200
Voltage (mV)
B
Outward Current (pA) at + 100mV
ns
ns
50
40
30
20
10
0
C
Inward Current (pA) at -100mV
ns
ns
0
-5
-10
-15
-20
-25
D
$I_T/I_{TMAX}$
1.0
0.5
0.0
-200
-100
0
100
200
Voltage (mV)

**Supplementary figure 4**

**Application of 100µM of Menthol, with extracellular calcium alone, did not alter hTRPA1 p.Ala172Val nor p.Asn855Ser channel excitability**.

Voltage-dependence activation of hTRPA1 p.Ala172Val and p.Asn855Ser was measured in response to Menthol. Current-voltage curves were measured with a two voltage-step protocol (voltage-ramps ranging from -100 mV to + 100 mV for 500 ms, every 5 seconds; holding potential was set at -70 mV).

A) Current densities were not statistically different for the variant channels when compared to WT as quantified in b) and c)
B) Outward current (at +100mV, WT = 13.29 ± 1.69pA/pF, p.Ala172Val = 17.51 ± 2.60pA/pF, p.Asn855Ser = 20.66 ± 3.35pA/pF were not significantly different ($p = 0.44$, and $p = 0.15$, respectively).
C) Inward currents (at -100mV, WT = -6.02 ± 1.11pA/pF, p.Ala172Val = -7.66 ± 1.83pA/pF, p.Asn855Ser = -7.49 ± 1.53pA/pF) were not significantly different ($p = 0.72$, $p = 0.80$, respectively).
D) Half-maximal activation voltage for WT n = 8, p.Ala172Val n = 8, and p.Asn855Ser n = 7 ($V_{1/2}$, WT= 65.39 ± 5.79mV, p.Arg185His= 56.61 ± 9.25mV, p.Asn855Serp.Asn855Ser= 58.62 ± 7.31mV, $p = 0.69$, and $p = 0.83$, respectively) were not statistically different. Slopes of the voltage-activation curve (k = 56.66 ± 1.40mV WT, 49.01 ± 1.53mV p.Arg185His, 49.73 ± 3.28mV p.Asn855Ser, $p= 0.022$ and $p = 0.069$, respectively) were reduced for both p.A712V and p.Asn855Ser .

WT – Wild Type
Data are presented as mean ± SEM.
Statistical analysis for group comparisons - One way ANOVA with Sidak's multiple comparison test (* - indicates statistically significant differences, $P <0.05$).

Supplementary table 1

| No | Clinical phenotype | Neuropathic pain grading | Gene | Nucleotide change | Amino acid change | Assigned pathogenicity | Alle frequency of variants in databases | | | | In silico analysis | | Previous study demonstrating altered channel function (PMID Reference) |
|---|---|---|---|---|---|---|---|---|---|---|---|---|---|
| | | | | | | | gnomAD | WGS10K | NPD European (African) | NIHR Bioresources controls European (African) | SIFT score | Polyphen | |
| 1 | Erythromelalgia | Definite | SCN9A | c.2543T>C | p.Ile848Thr | Pathogenic | . | 0.0001 | 0.0039 (0) | 0 (0) | 0.00 | probably damaging | Yes (15385606) |
| 2 | Erythromelalgia | Definite | SCN9A | c.2543T>C | p.Ile848Thr | Pathogenic | . | 0.0001 | 0.0039 (0) | 0 (0) | 0.00 | probably damaging | Yes (15385606) |
| 3 | Sensorimotor neuropathy | Probable | SPTLC1 | c.399T>G | p.Cys133Trp | Pathogenic | . | 0.0000 | 0.0039 (0) | 0 (0) | 0.00 | probably damaging | Yes (16210380) |
| 4 | Small fibre neuropathy | Definite | SCN10A | c.4984G>A | p.Gly1662Ser | Likely pathogenic | 0.0014 | 0.0010 | 0.0039 (0) | 0.0005 (0) | 0.00 | probably damaging | Yes (23115331) |
| 5 | Small fibre neuropathy | Definite | SCN11A | c.4628G>A | p.Cys1543Tyr | Likely pathogenic | 0.0001 | 0.00004 | 0.0039 (0.0131) | 0 (0) | 0.01 | probably damaging | No studies for this variant |
| 6 | Painful sensory neuropathy | Definite | SPTLC2 | c.886A>C | p.Ile296Leu | VUS | . | 0.0000 | 0.0039 (0) | 0 (0) | 0.04 | possibly damaging | No studies for this variant |
| 7 | Non-freezing cold injury | Definite | SCN9A | c.554G>A | p.Arg185His | VUS | 0.0031 | 0.0016 | 0 (0.0921) | 0.0015 (0.0064) | 0.01 | probably damaging | Yes (21698661, 22826602) |
| 8 | Non-freezing cold injury | Definite | SCN9A | c.554G>A | p.Arg185His | VUS | 0.0031 | 0.0016 | 0 (0.0921) | 0.0015 (0.0064) | 0.01 | probably damaging | Yes (21698661, 22826602) |
| 9 | Non-freezing cold injury | Definite | SCN9A | c.554G>A | p.Arg185His | VUS | 0.0031 | 0.0016 | 0 (0.0921) | 0.0015 (0.0064) | 0.01 | probably damaging | Yes (21698661, 22826602) |
| 10 | Non-freezing cold injury | Definite | SCN9A | c.554G>A | p.Arg185His | VUS | 0.0031 | 0.0016 | 0 (0.0921) | 0.0015 (0.0064) | 0.01 | probably damaging | Yes (21698661, 22826602) |
| 11 | Non-freezing cold injury | Definite | SCN9A | c.554G>A | p.Arg185His | VUS | 0.0031 | 0.0016 | 0 (0.0921) | 0.0015 (0.0064) | 0.01 | probably damaging | Yes (21698661, 22826602) |
| 12 | Non-freezing cold injury | Definite | SCN9A | c.554G>A | p.Arg185His | VUS | 0.0031 | 0.0016 | 0 (0.0921) | 0.0015 (0.0064) | 0.01 | probably damaging | Yes (21698661, 22826602) |
| 13 | Small fibre neuropathy | Definite | SCN9A | c.4612T>C | p.Trp1538Arg | VUS | 0.0020 | 0.0018 | 0.0039 (0) | 0.0018 (0) | 0.71 | benign | Yes (23292638) |
| 14 | Small fibre neuropathy | Definite | SCN9A | c.1445A>G | p.Lys482Arg | VUS | 0 | 0.00004 | 0.0039 (0) | 0 (0) | 0.18 | benign | No studies for this variant |
| 15 | Small fibre neuropathy | Probable | SCN9A | c.2215A>G | p.Ile739Val | VUS | 0.0024 | 0.0032 | 0.0078 (0) | 0.0041 (0) | 0.03 | benign | Yes (22826602) |
| 16 | Small fibre neuropathy | Definite | SCN9A | c.2215A>G | p.Ile739Val | VUS | 0.0024 | 0.0032 | 0.0078 (0) | 0.0041 (0) | 0.03 | benign | Yes (22826602) |
| 17 | Painful sensory neuropathy | Probable | SCN9A | c.2215A>G | p.Ile739Val | VUS | 0.0024 | 0.0032 | 0.0078 (0) | 0.0041 (0) | 0.03 | benign | Yes (22826602) |
| 18 | Sensorimotor neuropathy | Definite | SCN9A | c.2215A>G | p.Ile739Val | VUS | 0.0024 | 0.0032 | 0.0078 (0) | 0.0041 (0) | 0.03 | benign | Yes (22826602) |
| 19 | Small fibre neuropathy | Definite | SCN9A | c.4982A>G | p.Glu1661Gly | VUS | 0.0000 | 0.0001 | 0.0039 (0) | 0.0018 (0) | 0.10 | benign | No studies for this variant |
| 20 | Traumatic neuropathy | Probable | SCN9A | c.554G>A | p.Arg185His | VUS | 0.0031 | 0.0016 | 0 (0.0921) | 0.0015 (0.0064) | 0.01 | probably damaging | Yes (21698661, 22826602) |
| 21 | Painful Sensory neuropathy | Definite | SCN10A | c.3445G>A | p.Pro1149Met | VUS | 0.0001 | 0.0003 | 0.0039 (0) | 0.00008 (0) | 0.00 | probably damaging | No studies for this variant |
| 22 | Small fibre neuropathy | Definite | SCN10A | c.2428G>T | p.Gly810Trp | VUS | 0.0003 | 0.0005 | 0.0039 (0) | 0.0004 (0) | 0.00 | probably damaging | No studies for this variant |
| 23 | Painful Sensory neuropathy | Definite | SCN10A | c.2737G>A | p.Ala913Thr | VUS | 0.0003 | 0.0006 | 0.0039 (0) | 0.0003 (0) | 0.01 | probably damaging | No studies for this variant |
| 24 | Erythromelalgia | Probable | SCN10A | c.968A>G | p.Tyr323Cys | VUS | . | 0.00004 | 0.0039 (0) | 0 (0) | 0.00 | probably damaging | No studies for this variant |
| 25 | Small fibre neuropathy | Definite | SCN11A | c.2471A>G | p.Glu824Gly | VUS | . | 0.00004 | 0.0039 (0) | 0 (0) | 0.02 | possibly damaging | No studies for this variant |
| 26 | Episodic pain | Possible | SCN11A | c.1730C>T | p.Pro577Leu | VUS | 0.0002 | 0.0006 | 0.0039 (0) | 0.0005 (0) | 0.00 | probably damaging | No studies for this variant |

**Supplementary table 1**

List of gene variants that were reported and were deemed medically actionable i.e. variants that result in specific, defined medical recommendations. Clinically relevant, medically actionable, variants were reported to the referring clinician.

All participants were heterozygous for the relevant variants
All variants were predicted by Ensembl Variant Effect Predictor as missense variants
VUS – variant of uncertain significance
gnomAD - Genome Aggregation Database, open resource that aggregates and harmonises both exome and genome sequencing data; current status lists 125,748 exome sequences and 15,708 whole-genome sequences from unrelated individuals sequenced as part of various disease-specific and population genetic studies.
Allele Frequencies are shown in the Neuropathic Pain Disorders (NPD) cohort and in NIHR BioResource control cohorts.
WGS10K – NIHR Bioresources database with 13,037 whole genome sequences.
NPD- Neuropathic Pain Disorders
*In silico* analysis, SIFT and Polyphen, results shown.
SIFT score predicts whether an amino acid substitution affects protein function. The SIFT score ranges from 0.0 (deleterious) to 1.0 (tolerated).
The score can be interpreted as follows:
0.0 to 0.05 - Variants with scores in this range are considered deleterious. Variants with scores closer to 0.0 are more confidently predicted to be deleterious.
0.05 to 1.0 -Variants with scores in this range are predicted to be tolerated (benign). Variants with scores very close to 1.0 are more confidently predicted to be tolerated.

| Genomic coordinates and the HGNC gene symbol for gene models | Fraction with rare variants | Variants considered | P Value | Rho |
|---|---|---|---|---|
| **Tier 1-3 pain genes** | | | | |
| **Europeans** | | | | |
| **Probable/Definite Neuropathic pain vs Controls** | | | | |
| 1:212733740-213037331_FLVCR1_promoter | 0.078509 | 704 | 2.0274e-07 | 0 |
| 2:241656788-241737150_KIF1A | 0.038265 | 180 | 2.7235e-09 | 0 |
| 2:241757820-241808205_KIF1A_promoter | 0.01171 | 182 | 2.4975e-07 | |
| 1:115828713-115836247_NGF | 0.002639 | 14 | 2.6429e-09 | 0 |
| 12:52056606-52201160_SCN8A | 0.0097312 | 44 | 1.5018e-09 | 0 |
| 2:167227721-167351474_SCN9A_promoter | 0.037605 | 461 | 2.3805e-05 | 0 |
| 8:72935185-72987631_TRPA1 | 0.015009 | 73 | 5.2482e-05 | 0 |
| 2:234835229-234916724_TRPM8 | 0.017978 | 90 | 2.825e-07 | 0 |
| **Possible Neuropathic pain vs Controls** | | | | |
| 5:10237637-10311587_CCT5_promoter | 0.043829 | 390 | 2.1646e-10 | 0 |
| 22:19158112-19468896_CLTCL1_promoter | 0.12628 | 755 | 1.8541e-05 | 0 |
| 2:27272295-27888854_MPV17_promoter | 0.14408 | 1039 | 1.1537e-06 | 0 |
| 9:132888940-133713656_PRDM12_promoter | 0.12242 | 778 | 2.5937e-24 | 0 |
| 3:38887266-38992020_SCN11A | 0.022166 | 116 | 3.2814e-17 | 0 |
| 3:38387651-39150434_SCN11A_promoter | 0.036776 | 182 | 9.13e-36 | 0 |
| 2:167051740-167168308_SCN9A | 0.031066 | 138 | 1.0185e-11 | 0 |
| 9:94179481-95733983_SPTLC1_promoter | 0.17162 | 1253 | 1.4402e-20 | 0 |
| 14:77972340-78045374_SPTLC2 | 0.023678 | 113 | 7.4671e-17 | 0 |
| 17:16188184-16396312_TRPV2_promoter | 0.16927 | 1239 | 2.8037e-07 | 0 |
| 14:23990197-24004569_ZFHX2 | 0.037615 | 195 | 1.8008e-11 | 0 |
| **Idiopathic vs Controls** | | | | |
| 9:111694228-111884278_ELP1_promoter | 0.099514 | 665 | 0.00010639 | 0 |
| 1:212733740-213037331_FLVCR1_promoter | 0.078238 | 689 | 3.5673e-11 | 0 |
| 9:111637167-111693371_IKBKAP | 0.021612 | 98 | 6.0954e-14 | 0 |
| 2:27532377-27548436_MPV17 | 0.0065338 | 31 | 2.3621e-11 | 0 |
| 2:27272295-27888854_MPV17_promoter | 0.14492 | 1039 | 1.8079e-11 | 0 |
| 17:40688345-40696166_NAGLU | 0.0065338 | 31 | 2.5867e-11 | 0 |
| 1:156735601-156831188_NTRK1_promoter | 0.053443 | 606 | 1.2915e-12 | 0 |
| 3:38387651-39150434_SCN11A_promoter | 0.03669 | 181 | 7.875e-07 | 0 |
| 12:52056606-52201160_SCN8A | 0.0093818 | 42 | 7.6272e-10 | 0 |
| 2:167227721-167351474_SCN9A_promoter | 0.03736 | 445 | 3.7788e-07 | 0 |
| 9:94794766-94877614_SPTLC1 | 0.0073714 | 41 | 2.0265e-12 | 0 |
| 8:72935185-72987631_TRPA1 | 0.01491 | 72 | 2.1457e-10 | 0 |
| **Non-freezing cold injury vs Controls** | | | | |
| 11:62605687-64041758_ATL3_promoter | 0.2605 | 1807 | 1.9334e-26 | 0 |
| 22:19158112-19468896_CLTCL1_promoter | 0.12622 | 755 | 1.7755e-11 | 0 |
| 6:56322787-56765386_DST | 0.064706 | 326 | 7.2668e-21 | 0 |
| 2:241653211-241737219_KIF1A | 0.037479 | 174 | 2.6565e-24 | 0 |
| 2:27272295-27888854_MPV17_promoter | 0.14437 | 1036 | 2.418e-05 | 0 |
| 1:115828571-115836350_NGF | 0.002521 | 13 | 6.9657e-126 | 0 |
| 1:115257831-115881445_NGF_promoter | 0.073613 | 415 | 9.0969e-55 | 0 |
| 12:51791830-52209151_SCN8A_promoter | 0.073613 | 593 | 5.8951e-06 | 0 |
| 9:94179481-95733983_SPTLC1_promoter | 0.17143 | 1244 | 5.4476e-30 | 0 |
| 14:77768191-78083941_SPTLC2_promoter | 0.06084 | 683 | 1.1154e-26 | 0 |
| 2:234825742-235408070_TRPM8_promoter | 0.058824 | 421 | 9.7579e-49 | 0 |
| 12:109567961-110487716_TRPV4_promoter | 0.16185 | 898 | 1.6927e-12 | 0 |
| **Erythromelalgia vs Controls** | | | | |
| 5:10254263-10266494_CCT5 | 0.013754 | 63 | 1.1515e-09 | 0 |
| 19:9647679-11208553_DNMT1_promoter | 0.44918 | 3552 | 5.0184e-07 | 0 |
| 6:56322787-56765386_DST | 0.064743 | 328 | 9.2064e-09 | 0 |
| 9:111694228-111884278_ELP1_promoter | 0.099631 | 660 | 2.6704e-12 | 0 |
| 1:212733740-213037331_FLVCR1_promoter | 0.078665 | 695 | 4.5875e-62 | 0 |

| | | | | |
|---|---|---|---|---|
| 12:5020374-5027410_KCNA1 | 0.022979 | 114 | 8.8721e-09 | 0 |
| 20:43720954-43728938_KCNS1 | 0.016437 | 79 | 5.6901e-10 | 0 |
| 20:43593848-44003217_KCNS1_promoter | 0.046964 | 245 | 6.3713e-22 | 0 |
| 2:241653211-241737219_KIF1A | 0.037571 | 174 | 9.983e-40 | 0 |
| 2:27272295-27888854_MPV17_promoter | 0.14425 | 1040 | 4.0699e-07 | 0 |
| 1:115257831-115881445_NGF_promoter | 0.073465 | 414 | 4.0189e-19 | 0 |
| 3:38738320-38835432_SCN10A | 0.024992 | 117 | 2.745e-08 | 0 |
| 12:52078070-52202289_SCN8A | 0.0093928 | 42 | 6.9227e-19 | 0 |
| 2:167051740-167168308_SCN9A | 0.03103 | 138 | 0.00011167 | 0 |
| 17:75303232-75496662_SEPT9 | 0.020798 | 92 | 9.6699e-10 | 0 |
| 17:74347488-75883645_SEPTIN9_promoter | 0.68031 | 14656 | 2.0928e-23 | 0 |
| 9:94179481-95733983_SPTLC1_promoter | 0.17159 | 1249 | 1.658e-17 | 0 |
| 2:234835229-234928166_TRPM8 | 0.017612 | 88 | 5.975e-10 | 0 |
| 12:936533-1020561_WNK1 | 0.030191 | 130 | 9.9286e-06 | 0 |
| **Post Traumatic Neuropathy vs controls** | | | | |
| 14:50777141-51327966_ATL1_promoter | 0.094511 | 689 | 3.6407e-14 | 0 |
| 11:62605687-64041758_ATL3_promoter | 0.2607 | 1807 | 7.2586e-06 | 0 |
| 19:9647679-11208553_DNMT1_promoter | 0.44972 | 3553 | 2.0827e-11 | 0 |
| 1:46215085-47268057_FAAH_promoter | 0.10307 | 553 | 5.4233e-06 | 0 |
| 9:111629802-111693471_IKBKAP | 0.021655 | 96 | 1.8508e-18 | 0 |
| 20:43720954-43728938_KCNS1 | 0.016451 | 79 | 1.9211e-10 | 0 |
| 2:241653211-241737219_KIF1A | 0.037771 | 176 | 2.0856e-38 | 0 |
| 9:132888940-133713656_PRDM12_promoter | 0.12187 | 777 | 4.1157e-06 | 0 |
| 3:38387651-39150434_SCN11A_promoter | 0.036596 | 182 | 7.715e-09 | 0 |
| 12:51791830-52209151_SCN8A_promoter | 0.073527 | 596 | 4.1392e-20 | 0 |
| 2:234835229-234928166_TRPM8 | 0.017626 | 88 | 1.6971e-24 | 0 |
| 18:29077164-29267441_TTR_promoter | 0.077052 | 399 | 2.057e-07 | 0 |
| 12:678629-1060004_WNK1_promoter | 0.1296 | 1229 | 3.4161e-11 | 0 |
| **Neuropathic Pain Not Otherwise Specified vs controls** | | | | |
| 5:10237637-10311587_CCT5_promoter | 0.043807 | 390 | 1.7269e-05 | 0 |
| 6:56322787-56765386_DST | 0.064787 | 328 | 1.2338e-16 | 0 |
| 1:212733740-213037331_FLVCR1_promoter | 0.078214 | 689 | 3.2979e-10 | 0 |
| 1:115257831-115881445_NGF_promoter | 0.073347 | 411 | 5.1011e-05 | 0 |
| 1:156735601-156831188_NTRK1_promoter | 0.053206 | 606 | 4.9332e-07 | 0 |
| 9:132888940-133713656_PRDM12_promoter | 0.12219 | 777 | 5.1652e-10 | 0 |
| 3:38387651-39150434_SCN11A_promoter | 0.036925 | 183 | 1.1968e-45 | 0 |
| 12:52078070-52202289_SCN8A | 0.0093991 | 41 | 9.7448e-23 | 0 |
| 2:167051740-167168308_SCN9A | 0.031051 | 137 | 1.1055e-07 | 0 |
| 2:167227721-167351474_SCN9A_promoter | 0.037261 | 441 | 1.2761e-07 | 0 |
| 9:94179481-95733983_SPTLC1_promoter | 0.17153 | 1253 | 9.4963e-18 | 0 |
| 14:77972340-78045374_SPTLC2 | 0.023666 | 113 | 4.924e-13 | 0 |
| | | | | |
| **Africans** | | | | |
| **Post Traumatic Neuropathy vs controls** | | | | |
| 6:56323823-56819337_DST | 0.076923 | 14 | 8.3758e-05 | 0 |
| **Small Fibre Neuropathy vs controls** | | | | |
| 2:241653211-241737219_KIF1A | 0.044586 | 7 | 1.9638e-06 | 0 |
| 2:234835229-234928166_TRPM8 | 0.044586 | 7 | 6.7973e-06 | 0 |
| 12:936533-1020561_WNK1 | 0.031847 | 5 | 0.00010244 | 0 |
| 12:678629-1060004_WNK1_promoter | 0.17834 | 64 | 6.2972e-06 | 0 |
| **Non-freezing cold injury vs Controls** | | | | |
| 1:156811877-156851636_NTRK1 | 0.02139 | 4 | 7.1487e-09 | 0 |

**Supplementary table 2**
Significant results from the gene-wise association test for rare variants of Tier 1-3 genes for each ethnicity and phenotype. Significance was set to the Bonferroni adjusted 0.01 threshold for the number of genes considered (0.01/36 = 2.7*10e-4 ). We report unadjusted P values that passed the Bonferroni corrected significant threshold.

| Genomic coordinates and the HGNC gene symbol for gene models | Fraction with rare variants | Variants considered | P Value | Rho |
|---|---|---|---|---|
| **Whole gene set** | | | | |
| **Europeans** | | | | |
| **All Neuropathic pain vs Controls** | | | | |
| 16:16101815-16235133_ABCC1 | 0.0046106 | 13 | 3.57E-10 | 0 |
| 8:107773299-107782388_ABRA | 0.0024699 | 6 | 8.48E-11 | 0 |
| 7:6713448-6715965_AC073343.1 | 0.0016466 | 5 | 1.53E-07 | 0.1 |
| 14:70989304-70991552_ADAM20 | 0.0013173 | 4 | 6.73E-09 | 0.1 |
| 7:87563806-87822444_ADAM22 | 0.00098798 | 3 | 7.66E-09 | 0.1 |
| 5:178540879-178771007_ADAMTS2 | 0.0024699 | 6 | 2.42E-10 | 0 |
| 1:26648464-26680526_AIM1L | 0.0062572 | 15 | 9.11E-09 | 0 |
| 10:45869747-45941096_ALOX5 | 0.001976 | 5 | 1.02E-12 | 0 |
| 2:242128052-242163531_ANO7 | 0.0027993 | 8 | 1.72E-12 | 0 |
| 12:99042160-99126330_APAF1 | 0.0039519 | 9 | 1.93E-08 | 0 |
| 2:68694871-68805137_APLF | 0.0011526 | 3 | 1.26E-14 | 0 |
| 1:94639450-94697137_ARHGAP29 | 0.0034579 | 9 | 6.86E-09 | 0 |
| 10:28067822-28287922_ARMC4 | 0.0049399 | 13 | 5.40E-08 | 0 |
| 19:42470756-42492732_ATP1A3 | 0.0021406 | 6 | 3.93E-13 | 0 |
| 9:33255285-33264660_BAG1 | 0.0013173 | 4 | 3.90E-07 | 0.1 |
| 15:52402005-52404915_BCL2L10 | 0.0011526 | 3 | 2.72E-15 | 0 |
| 5:78373352-78384356_BHMT2 | 0.0024699 | 5 | 4.47E-08 | 0 |
| 17:41197711-41276095_BRCA1 | 0.0042812 | 11 | 4.88E-10 | 0 |
| 11:82625764-82645231_C11orf82 | 0.0011526 | 3 | 1.53E-15 | 0 |
| 12:70272858-70352309_C12orf28 | 0.001976 | 4 | 9.64E-10 | 0 |
| 14:105052787-105055181_C14orf180 | 0.0023053 | 7 | 1.25E-15 | 0 |
| 12:7246446-7261770_C1RL | 0.0029639 | 7 | 2.59E-12 | 0.1 |
| 3:185431276-185447460_C3orf65 | 0.001976 | 3 | 1.54E-08 | 0 |
| 15:42652136-42704352_CAPN3 | 0.0026346 | 5 | 8.84E-08 | 0 |
| 1:10699139-10725481_CASZ1 | 0.0051046 | 14 | 2.25E-10 | 0 |
| 19:47761828-47774873_CCDC9 | 0.00098798 | 3 | 2.06E-10 | 0.1 |
| 11:35160867-35250840_CD44 | 0.001482 | 5 | 9.59E-10 | 0.1 |
| 8:42559625-42591717_CHRNB3 | 0.00098798 | 3 | 1.23E-09 | 0.1 |
| 20:61926481-61960987_COL20A1 | 0.0065865 | 15 | 3.79E-07 | 0 |
| 19:15752299-15770077_CYP4F3 | 0.0029639 | 7 | 2.24E-07 | 0 |
| 2:118575082-118588191_DDX18 | 0.0011526 | 3 | 6.20E-08 | 0.2 |
| 6:170592107-170598890_DLL1 | 0.0016466 | 3 | 2.16E-10 | 0.3 |
| 5:169081464-169509843_DOCK2 | 0.0021406 | 6 | 4.76E-14 | 0 |
| 19:2189756-2227118_DOT1L | 0.0059279 | 16 | 1.15E-08 | 0 |
| 1:44435905-44438206_DPH2 | 0.0013173 | 4 | 1.75E-09 | 0.1 |
| 16:22237076-22295307_EEF2K | 0.0031286 | 8 | 2.20E-10 | 0 |
| 12:10658548-10675292_EIF2S3L | 0.001976 | 6 | 3.11E-15 | 0 |
| 6:132129287-132211535_ENPP1 | 0.0032933 | 7 | 3.99E-07 | 0 |
| 12:15774255-15835855_EPS8 | 0.0018113 | 4 | 8.99E-11 | 0 |
| 8:614610-681152_ERICH1 | 0.0027993 | 6 | 3.62E-09 | 0 |
| 3:38537860-38583424_EXOG | 0.0029639 | 7 | 1.93E-07 | 0.1 |
| 4:91229615-92520132_FAM190A | 0.0023053 | 4 | 6.81E-09 | 0 |
| 15:52874400-52944065_FAM214A | 0.0016466 | 5 | 1.28E-07 | 0.1 |
| 18:55217969-55253816_FECH | 0.00098798 | 3 | 2.06E-09 | 0.1 |
| 9:95738584-95797806_FGD3 | 0.0027993 | 7 | 1.26E-15 | 0 |
| 6:76005652-76124663_FILIP1 | 0.0029639 | 7 | 6.31E-08 | 0 |
| 9:14737440-14868953_FREM1 | 0.0090565 | 25 | 1.29E-07 | 0 |
| 21:47556381-47575416_FTCD | 0.0034579 | 7 | 1.94E-08 | 0 |
| 6:46821758-46874402_GPR116 | 0.0027993 | 7 | 8.74E-10 | 0 |

| | | | | |
|---|---|---|---|---|
| 6:142630695-142764637_GPR126 | 0.0034579 | 9 | 9.52E-11 | 0 |
| 17:72363674-72368735_GPR142 | 0.001976 | 5 | 1.89E-24 | 0.1 |
| 2:128407614-128409316_GPR17 | 0.0016466 | 4 | 2.22E-10 | 0 |
| 19:35500042-35517022_GRAMD1A | 0.0049399 | 12 | 1.15E-07 | 0.1 |
| 4:2965737-3040173_GRK4 | 0.0013173 | 4 | 2.68E-18 | 0 |
| 22:46693331-46725456_GTSE1 | 0.0044459 | 11 | 6.63E-08 | 0 |
| 14:25075829-25078803_GZMH | 0.00098798 | 3 | 4.47E-10 | 0.1 |
| 17:65074393-65214916_HELZ | 0.0042812 | 11 | 1.12E-08 | 0 |
| 11:93754556-93845053_HEPHL1 | 0.0037873 | 10 | 7.87E-10 | 0 |
| 3:42734320-42742317_HHATL | 0.0034579 | 7 | 2.21E-07 | 0 |
| 9:133260072-133309076_HMCN2 | 0.0047752 | 11 | 1.44E-08 | 0 |
| 2:203644274-203735380_ICA1L | 0.0018113 | 4 | 4.20E-18 | 0.2 |
| 3:129159189-129239058_IFT122 | 0.0026346 | 7 | 2.42E-10 | 0 |
| 1:67786085-67861620_IL12RB2 | 0.001976 | 6 | 2.88E-07 | 0 |
| 6:52101751-52109225_IL17F | 0.00098798 | 3 | 1.15E-09 | 0.1 |
| 4:128554197-128637586_INTU | 0.00098798 | 3 | 8.96E-09 | 0.2 |
| 1:156496351-156539238_IQGAP3 | 0.0057632 | 13 | 4.16E-08 | 0 |
| 16:54317544-54319854_IRX3 | 0.0023053 | 6 | 7.95E-13 | 0 |
| 17:45331244-45412723_ITGB3 | 0.001482 | 4 | 3.51E-14 | 0 |
| 14:93407968-93581414_ITPK1 | 0.001482 | 4 | 1.57E-08 | 0.1 |
| 11:64060618-64067210_KCNK4 | 0.0013173 | 4 | 6.24E-18 | 0 |
| 6:73331924-73905055_KCNQ5 | 0.001482 | 4 | 7.64E-17 | 0.1 |
| 2:241656788-241737150_KIF1A | 0.0069159 | 14 | 2.02E-08 | 0 |
| 5:132032369-132073108_KIF3A | 0.0016466 | 3 | 1.51E-09 | 0 |
| X:69510002-69640098_KIF4A | 0.0011526 | 3 | 7.75E-08 | 0.1 |
| 19:55294383-55295260_KIR2DL1 | 0.0024699 | 5 | 7.74E-12 | 0.1 |
| 11:126294495-126870380_KIRREL3 | 0.0029639 | 8 | 2.96E-16 | 0 |
| 3:189675726-189838468_LEPREL1 | 0.0026346 | 7 | 6.34E-12 | 0 |
| 7:123296025-123303794_LMOD2 | 0.00098798 | 3 | 2.08E-10 | 0.1 |
| 10:85981710-85985273_LRIT2 | 0.00098798 | 3 | 1.15E-10 | 0 |
| 21:30303571-30365237_LTN1 | 0.0032933 | 9 | 1.07E-09 | 0 |
| 19:43965538-43969718_LYPD3 | 0.0013173 | 4 | 3.80E-09 | 0.1 |
| 15:100269338-100272192_LYSMD4 | 0.0011526 | 3 | 3.91E-12 | 0 |
| 10:102762320-102766905_LZTS2 | 0.001482 | 4 | 1.51E-15 | 0 |
| 5:71403450-71500986_MAP1B | 0.0046106 | 12 | 6.24E-11 | 0 |
| 14:71197219-71267779_MAP3K9 | 0.0037873 | 8 | 5.99E-08 | 0 |
| 9:123367518-123476611_MEGF9 | 0.0026346 | 5 | 1.43E-07 | 0 |
| 17:81042826-81052312_METRNL | 0.0018113 | 4 | 2.64E-10 | 0 |
| 17:45901575-45908862_MRPL10 | 0.0013173 | 3 | 6.88E-13 | 0 |
| 19:54376827-54377745_MYADM | 0.0023053 | 5 | 5.80E-11 | 0 |
| 17:10532922-10558356_MYH3 | 0.0037873 | 10 | 1.11E-12 | 0 |
| 14:23851265-23876383_MYH6 | 0.0023053 | 7 | 2.96E-07 | 0 |
| 5:177546645-177548999_N4BP3 | 0.0018113 | 5 | 6.64E-10 | 0.1 |
| 1:46049820-46083784_NASP | 0.0018113 | 5 | 4.33E-17 | 0 |
| 4:17812738-17844936_NCAPG | 0.0021406 | 5 | 2.13E-10 | 0 |
| 8:71033608-71128953_NCOA2 | 0.0024699 | 6 | 2.64E-10 | 0 |
| 17:26084316-26125784_NOS2 | 0.0034579 | 7 | 1.73E-10 | 0 |
| 8:27880856-27931905_NUGGC | 0.0021406 | 6 | 3.02E-12 | 0 |
| 15:41625208-41672367_NUSAP1 | 0.0016466 | 5 | 2.98E-08 | 0.1 |
| 5:140797470-140890735_PCDHGB7 | 0.0036226 | 9 | 1.59E-10 | 0 |
| 1:77558163-77685087_PIGK | 0.0011526 | 3 | 5.37E-15 | 0 |
| 19:39904703-39915987_PLEKHG2 | 0.0054339 | 13 | 1.01E-07 | 0 |
| 14:65194318-65210406_PLEKHG3 | 0.0077392 | 17 | 2.78E-08 | 0 |
| 19:4504520-4517590_PLIN4 | 0.0016466 | 5 | 9.03E-08 | 0.1 |
| 9:33750530-33799176_PRSS3 | 0.0018113 | 4 | 8.90E-11 | 0 |
| 16:58314167-58327720_PRSS54 | 0.0026346 | 6 | 5.24E-09 | 0 |

| | | | | |
|---|---|---|---|---|
| 5:71616283-71654214_PTCD2 | 0.0011526 | 3 | 3.34E-07 | 0.2 |
| 6:47846053-48036289_PTCHD4 | 0.001482 | 4 | 9.82E-08 | 0 |
| 11:450550-490493_PTDSS2 | 0.0023053 | 5 | 1.86E-20 | 0.1 |
| 3:110790965-110912153_PVRL3 | 0.001976 | 5 | 1.49E-12 | 0 |
| 6:107077557-107113791_QRSL1 | 0.0023053 | 7 | 2.22E-07 | 0 |
| 16:12181701-12183753_RP11-276H1.3 | 0.0011526 | 3 | 2.13E-14 | 0 |
| 20:62190630-62203698_RP4-697K14.7 | 0.011856 | 30 | 2.07E-12 | 0 |
| 5:115782325-115840589_SEMA6A | 0.0052692 | 13 | 1.56E-07 | 0.1 |
| 15:44087238-44092184_SERINC4 | 0.00098798 | 3 | 3.07E-09 | 0.1 |
| 5:150696439-150727020_SLC36A2 | 0.001976 | 5 | 4.05E-11 | 0 |
| 9:115642055-115652961_SLC46A2 | 0.0016466 | 5 | 3.02E-07 | 0.1 |
| 4:139093129-139163217_SLC7A11 | 0.0021406 | 4 | 2.47E-09 | 0 |
| 2:231305272-231407567_SP100 | 0.001976 | 6 | 6.37E-08 | 0 |
| 7:99913463-99917393_SPDYE3 | 0.0011526 | 3 | 7.63E-14 | 0 |
| 2:228846486-229046289_SPHKAP | 0.0046106 | 13 | 8.40E-12 | 0 |
| 17:79977081-79980514_STRA13 | 0.0011526 | 3 | 4.76E-14 | 0 |
| 3:33194278-33260276_SUSD5 | 0.0021406 | 5 | 1.19E-07 | 0.2 |
| 16:29989477-30002833_TAOK2 | 0.0031286 | 8 | 8.22E-11 | 0 |
| 1:150460442-150479509_TARS2 | 0.0018113 | 6 | 9.51E-12 | 0 |
| 9:100961748-101017784_TBC1D2 | 0.0021406 | 6 | 5.01E-15 | 0 |
| 4:48139449-48230531_TEC | 0.0013173 | 4 | 1.08E-08 | 0.1 |
| 22:37387239-37398303_TEX33 | 0.001482 | 4 | 8.23E-15 | 0 |
| 7:128597331-128694794_TNPO3 | 0.0026346 | 6 | 2.01E-12 | 0 |
| 9:15172019-15307233_TTC39B | 0.0013173 | 4 | 8.90E-08 | 0.1 |
| 17:46846397-46894432_TTLL6 | 0.0039519 | 9 | 3.56E-09 | 0 |
| 16:11773077-11836586_TXNDC11 | 0.0052692 | 12 | 2.97E-09 | 0 |
| 2:128848939-128945123_UGGT1 | 0.0039519 | 12 | 1.56E-29 | 0 |
| 2:61415248-61647956_USP34 | 0.0064219 | 15 | 3.39E-21 | 0 |
| 9:136629191-136857358_VAV2 | 0.0021406 | 5 | 1.25E-08 | 0 |
| 19:54544235-54567012_VSTM1 | 0.0021406 | 4 | 2.39E-08 | 0 |
| 7:141408701-141430098_WEE2 | 0.00098798 | 3 | 1.61E-08 | 0.1 |
| 19:15533906-15559106_WIZ | 0.0055986 | 14 | 3.82E-22 | 0 |
| 1:155629494-155658171_YY1AP1 | 0.0024699 | 7 | 2.34E-15 | 0 |
| 4:25314444-25370677_ZCCHC4 | 0.0024699 | 6 | 3.46E-09 | 0 |
| 5:32355972-32444381_ZFR | 0.0011526 | 3 | 2.82E-11 | 0.3 |
| 20:62339945-62367201_ZGPAT | 0.0021406 | 6 | 2.54E-10 | 0 |
| 19:20295169-20308907_ZNF486 | 0.0013173 | 4 | 5.17E-19 | 0 |
| 19:52937314-52954724_ZNF534 | 0.0016466 | 5 | 5.70E-08 | 0.1 |
| 22:24086024-24092897_ZNF70 | 0.0016466 | 5 | 1.22E-18 | 0 |
| 16:31446173-31448153_ZNF843 | 0.00098798 | 3 | 1.48E-08 | 0.1 |
| 10:126631147-126673505_ZRANB1 | 0.0013173 | 4 | 5.25E-08 | 0.1 |

**Supplementary table 3**

Significant results from the gene-wise association test for the whole gene set (5790). For the whole gene set analysis we applied a minor allele count filter of >= 2 and for the Neuropathic Pain Disorders candidate genes a minor allele count filter of >= 1. Only genes with at least two variants which passed the filters were included.

Significance was set to the Bonferroni adjusted 0.01 threshold for the number of genes considered (0.01/5790 = 1.7*10e-6).
